# Novel Web-Based Technology to Promote Goal-Setting in Complex Chronic Illness: Randomized Controlled Trial

**DOI:** 10.2196/70402

**Published:** 2026-02-20

**Authors:** Jody Lin, Bernd Huber, Ofra Amir, Shiri Assis-Hassid, Sebastian Gehrmann, Krzysztof Gajos, Barbara Grosz, Lee Sanders

**Affiliations:** 1 Department of Pediatrics, School of Medicine, University of Utah, 100 Mario Capecchi Drive, Salt Lake City, UT, 84114, United States, 1 8016623645; 2 Department of Computer Science, John A. Paulson School of Engineering and Applied Sciences (Computer Science), Harvard University, Cambridge, MA, United States; 3 Department of Data and Decision Sciences, Technion – Israel Institute of Technology, Haifa, Israel; 4 Department of Pediatrics, School of Medicine, Stanford University, Palo Alto, CA, United States

**Keywords:** children with medical complexity, digital health, family-centered care, chronic care, care coordination

## Abstract

**Background:**

Shared goal-setting is a common feature of quality guidelines to improve care quality for children with medical complexity, but few studies have examined the efficacy of interventions designed to improve goal-setting.

**Objective:**

This study aims to evaluate a novel internet-based tool to promote shared goal-setting (GoalKeeper) in the care of children with medical complexity.

**Methods:**

We conducted a randomized stepped-wedge trial (intervention vs usual care) between April 1, 2019 and March 21, 2021, at primary and subspecialty care clinics at an academic medical center, including 11 medical providers (medical doctor, doctor of osteopathic medicine, or nurse practitioner). Adult parents of children with medical complexity were eligible if they were English-speaking, with home internet access, and with children with medical complexity aged younger than 12 years. Participants were clustered by provider, with providers crossing over from control to intervention at different stages. The assignment of timing for crossover was random. Control group participants received usual care without any additional interventions. Intervention group participants received a novel web-based tool called GoalKeeper for initial use during the clinic visit and subsequent continued use after the clinic visit. GoalKeeper was co-designed by parents, providers, and computer scientists to include 2 modules, meant to be used by both the parent and medical provider for each child: (1) goal elicitation, used synchronously during a clinic visit; (2) tracking, used asynchronously between visits by parents and providers of children with medical complexity. The primary outcome was quality of goal-setting assessed by the Patient Assessment of Care for Chronic Illness Care goal-setting domain at baseline (t1) and 1 month (t2). We conducted a repeated-measures mixed-effects ANOVA to evaluate between- and within-group differences over time for fixed effects (timing of intervention, intervention×time) and random effect (provider cluster).

**Results:**

We enrolled 67 parent-child dyads (control: n=32 control; intervention: n=35). Parents had a mean age of 37.4 (SD 8.2) years, children with medical complexity with mean age 5.6 (SD 0.5) years, and 29 (44%) parents identified as Hispanic. Of the 35 intervention parents, 34 successfully used GoalKeeper during the clinical encounter with their provider. During the follow-up period, quality of goal-setting was sustained at t2 for the intervention group but declined for the control group (δ=0.03 vs −0.43; *F_49_*=3.52, *P*=.06). Similar patterns were observed for care quality (δ=0.01 vs −0.48; *F*_49_=4.28, *P*=.04).

**Conclusions:**

Our study demonstrates that family-centered goal-setting may help combat the gradual decline in care quality otherwise experienced by children with medical complexity in between clinic visits. Successful use of the tool with providers in clinic suggests that digital tools are feasible interventions to change family-provider communication around family goals.

## Introduction

Children with medical complexity and their families face many challenges. Defined by high service needs, high resource use, and functional disability, children with medical complexity represent less than 5% of all children but a disproportionately high share of pediatric care use, coordinated across multiple health care systems, providers, and agencies [1-4]. This complex ecosystem of care hinders caregivers’ and providers’ abilities to have a shared understanding of care priorities to effectively address health needs as a team. Moreover, the fluctuating nature of health issues faced by children with medical complexity results in constant changes to the care team, and helping new team members be up-to-date is highly challenging. Thus, parents of children with medical complexity are less likely to report high care quality and shared decision-making during ambulatory care encounters than parents of children with noncomplex chronic conditions [5-7].

To improve care quality and shared decision-making for children with medical complexity, several national pediatric organizations have suggested the importance of a family-centered care framework centered around shared goal-setting to create individualized care plans [8,9]. While prior studies have used multidisciplinary teams for individualized care plan creation, few models exist for effective and scalable tools that facilitate shared goal-setting when prolonged visits with highly resourced teams are unavailable [10,11]. Studies of noncomplex chronic conditions in pediatrics (eg, asthma or type 1 diabetes) suggest that technology-based tools may provide efficacious ways to establish and track goals during and between ambulatory care visits [12-14]. These tools result in improved satisfaction, reduced urgent care visits, and improved outcomes. However, technology-based solutions for the care of children with medical complexity must overcome unique challenges such as team hierarchies, loosely coupled teams (eg, most providers care for children with medical complexity in diffuse teams rather than tightly coordinated team-based clinics), and asynchronous time scales among providers [15]. Prior work suggests that shared care plans for children with medical complexity require common goals to be set synchronously by the provider and caregiver, then tracked asynchronously by both but has not previously been tested [15]. It is unknown whether technology-based tools can be effective in improving the care of children with medical complexity.

In this study, we evaluate the effectiveness of a technology-based tool for eliciting and tracking shared goals in the ambulatory care of children with medical complexity. Working in a multimodal development team comprised of health care providers, behavioral scientists, computer scientists, and parents of children with medical complexity, we developed a novel, health-information technology platform, called GoalKeeper, to improve goal-concordant conversations in real-world, outpatient care settings. We hypothesized that parents of children with medical complexity exposed to GoalKeeper would report higher care quality and higher quality goal-setting when compared to parents who received usual care.

## Methods

### Study Design

We conducted a prospective, stepped-wedge, randomized controlled trial of intervention efficacy. The study was conducted at primary and subspecialty ambulatory clinics associated with a children’s hospital at an academic medical center. In the stepped-wedge design, each provider first spent at least 3 weeks in the control condition, when research staff recruited parent-child dyads from their clinic to be in the control group, followed by crossover into the intervention group for 3 weeks. Providers crossed over at different intervals spaced 3 weeks apart (Figure S1 in Multimedia Appendix 1). The order that providers crossed over was randomized in R Statistical Software (v.3.5.3 R Core Team 2019) via RStudio (v.1.1.463 Posit team 2019) by author JL, with allocation and enrollment conducted by research assistants [16]. Each parent-child dyad served in either the control or intervention group, with the control group receiving usual care. We chose a stepped-wedge design because it offers the most feasible and effective approach to randomization of the parent-child dyads without introducing bias in the control group by changing provider behavior through exposure to the intervention [17]. Ethically, stepped-wedge designs are better suited for studies in which true equipoise has not been established for the interventions being tested [18]. Prior interviews with providers of children with medical complexity found that most providers were familiar with the practice of goal-setting and endorsed some level of goal-setting in their current practice, but structured approaches to goal-setting were not part of routine practice in these clinics [15]. For this study, we adhered to the CONSORT guidelines for reporting randomized clinical trials (see Checklist 1).

### Study Population

We recruited medical providers (medical doctor, doctor of osteopathic medicine, nurse practitioner, and physician assistant) from 2 ambulatory clinics that see a high proportion of children with medical complexity—pediatric neurology and complex primary care clinics (CPCC) at the main location of a single academic health center. The health center is part of a standalone tertiary children’s health system in northern California. All providers that were not trainees at these clinics were eligible for recruitment. We recruited a convenience sample of primary caregivers (eg, parents) of the patients seen by enrolled providers in person at the time of their clinic visit. We capped enrollment per provider cluster at 10 parents. Parents were eligible if they were aged 18 years or older, English-speaking, and with a child with medical complexity aged younger than 12 years. We excluded parents without home internet access or with known neurocognitive or psychiatric issues that precluded the ability to offer informed consent. We excluded older children, who may have the capacity to participate in decision-making, since the intervention was not designed for interaction with children [19]. We defined medical complexity as meeting each of 2 criteria in the 12 months prior to study enrollment: (1) ambulatory visits with at least 2 different subspecialties affiliated with the medical center and (2) medical fragility due to technology dependence (gastrostomy tube, nasogastric tube, ventriculoperitoneal shunt, or tracheostomy) or clinic visits at CPCC. Enrollment occurred between April 1, 2019 and December 21, 2020, with follow-up through March 21, 2021. Due to institutional restrictions prompted by the COVID-19 pandemic prohibiting in-person research activities that were necessary for study activities at enrollment, recruitment was paused between March 13, 2020 and July 20, 2020.

### Intervention

We designed a technology-based family-centered goal-setting tool (GoalKeeper) using iterative feedback from parents and providers of children with medical complexity and informed by the challenges of technology-based tools elucidated in our prior work [15]. In the first phase of the design process, we interviewed parents and providers to develop a taxonomy of goals and effective approaches to discussing goals in an outpatient clinic visit, based on prior frameworks for goal-concordant, chronic-illness care [20-22]. In the second phase, we conducted iterative user testing with parents and providers initially via paper-based prototypes and subsequently a technology-based version. We chose to host the intervention on the internet and to function outside of the electronic health record (EHR) to allow for rapid iteration of the intervention design and ease of access to the intervention from any device (eg, computer, tablet, or smartphone). We designed GoalKeeper using responsive web technology that optimizes its use for any device. This functionality enables parents and providers to quickly input data and access GoalKeeper in any setting with internet independent of the type of device used.

**Figure 1.**
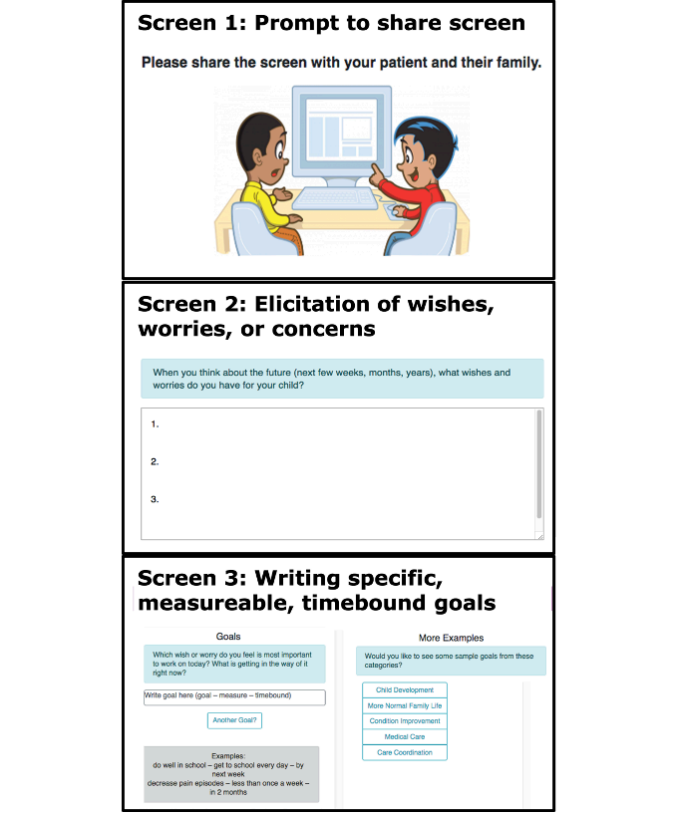
GoalKeeper goal elicitation module.

GoalKeeper consists of 2 separate modules: (1) Goal Elicitation and (2) Tracking. The key module and the focus of this study is the goal elicitation module, which was designed to be used in real time in the exam room, facilitating a conversation between the parent and the medical provider during a routine, nonurgent care visit, consists of 3 components, as shown in Figure 1. The first component provides prompts to elicit the parent’s “wishes/worries/concerns” about their child’s health. The second component helps the parent and provider derive from these “wishes/worries/concerns” at least 1 goal that is specific, measurable, and time-bound to ensure that the goals are actionable and attainable. Specific actions were not entered into the module to reduce documentation burden based on feedback during design interviews and pretesting. The third component provides sample goals as inspiration based on a taxonomy of common children with medical complexity goals developed in the design process (Assis-Hassid S et al, unpublished data, 2021). These 3 components are synthesized on the final screen, with a “copy” button to facilitate easy transfer (pasting) of the text into the EHR. The tracking module was designed to be set up by a provider immediately after the clinical encounter. The purpose of the tracking module was to address parents’ desires for symptoms tracking in the tool but was not the focus of the GoalKeeper design process given the prevalence of symptom tracking technology already in existence. The tracking module provides customizable templates that providers can assign to parents to track their progress toward the elicited goal(s). To allow for asynchronous work outside of clinic visits, parents and providers had distinct interfaces. Providers could create goals, create and assign tracking templates, and view data while parents could view set goals and view and input data into tracking templates. We placed goal elicitation in the provider interface, based on user feedback, to help flatten the parent-provider hierarchy in the clinic visit. GoalKeeper requires the medical provider to elicit and type the parent’s stated goals during the clinic visit. Prior prototypes that required parents to type their goals resulted in increased parental stress and decreased medical provider engagement. In contrast to usual practice, medical providers welcomed the during-encounter goal elicitation feature as an opportunity to engage more meaningfully with parents. We optimized the tracking module for use on smartphones or tablets based on parent feedback. During the trial, further development was frozen to ensure all participants were exposed to the same version of the intervention.

The intervention implementation process was developed and evaluated using the Consolidated Framework for Implementation Research (CFIR) [23]. Prior to the start of the trial, we conducted stakeholder focus groups using the CFIR companion interview guide to inform implementation of GoalKeeper that resulted in the selection of 3 implementation strategies: educational materials, individual technical assistance, and automated reminders [24]. Educational materials included paper and electronic copies of an instructional manual on GoalKeeper, with parent and provider versions. Parents received a 5-minute in-person overview of GoalKeeper while providers received a 30-minute orientation, including a 5-minute educational video [25]. The study team was available for in-person and remote technical assistance but did not attend clinic visits with participants. Parents received automated weekly email reminders to track their progress on goals. Details on the implementation process and evaluation can be found in a companion study [26].

Participants in the intervention group were given GoalKeeper to use during their initial clinic visit immediately after enrolling in the study. They created a unique login and password and were emailed the webpage link to access GoalKeeper on their own device. Study team members also accessed the GoalKeeper webpage on the clinic room computer immediately prior to the start of the participant’s clinic visit. Participants in the control group received usual clinical care with their provider.

### Measures and Outcomes

Our randomized controlled trial is a hybrid type I effectiveness-implementation trial with a primary focus on assessing the effectiveness of the intervention but included collection of preliminary data on barriers and facilitators to implementation, reported in a separate manuscript [26,27]. Measures of primary and secondary outcomes were collected electronically immediately after the initial clinical encounter for both parents and providers following study enrollment (T1) and at 1-month post-encounter (T2). Additional implementation outcome survey measures and exit interviews were collected at 3 months post-encounter (T3) for parents and, for providers, at the end of the study period for their last enrolled parent (Figure S2 in Multimedia Appendix 1). All measures were collected via secure electronic survey tools and stored electronically via the Stanford REDCap (Research Electronic Data Capture).

The primary outcome was the difference in parent-reported quality of goal-setting between the control and intervention groups from T1 to T2, as measured by adapted questions from the goal-setting domain of the Patients Assessment of Chronic Illness Care (PACIC), a 20-item (5-point Likert scale: no/never to yes/always) validated survey measure of chronic illness care quality based on the Chronic Care Model [28]. A mean composite score is calculated for each domain and the entire survey based on relevant items. The goal-setting domain contains 5 items that cover healthy behaviors, treatment plans, and community integration. We adapted this measure for children with medical complexity because of its wide use in assessing chronic illness care quality and the lack of alternate, validated, pediatric-specific measures. Secondary outcomes measure included the PACIC subdomains for problem-solving, care coordination, and composite outcome of care quality based on all measured PACIC subdomains.

Additional secondary outcome measures included quality of shared decision-making, quality of parent-provider communication, and parenting stress. We assessed quality of shared decision-making (T1 and T2) using the National Survey for Children with Special Health Care Needs 2009‐2010 shared decision-making domain, which creates a binary composite variable for high versus low quality shared decision-making from 4 survey questions at T1 and T2 [29]. We assessed the quality of parent-provider communication using the Communication Assessment Tool (T1), a validated 15-item (5-point Likert scale: 1-poor to 5-excellent) survey measure with mean composite outcome [30]. We assessed parenting stress using the Parenting Distress domain of the Parenting Stress Index (T1), which captures parent stress levels related to partner conflicts, social support, and life restrictions due to child rearing [31]. Scores are presented as a sum of all answers (range 12‐60) with higher scores indicating higher stress.

### Intervention Fidelity

We assessed intervention fidelity and quality in 2 ways: (1) capture of information directly from the GoalKeeper platform and (2) participant interviews at T3. Within the app, we primarily assessed number of goals set per intervention group participant but also assessed frequency of app use, and counts of data entered into each field. Through semistructured interviews, we assessed barriers and facilitators to implementation using questions from the CFIR companion interview guide (Multimedia Appendix 2), detailed in a separate manuscript [26,32].

### Sample Size

We aimed to identify a moderate change in the PACIC domain of goal-setting from T1 to T2. Prior studies of the PACIC have shown in the domain of goal-setting a mean of 2.43 out of 5 (SD 1.1) [33]. We anticipated at least a δ of 0.5 (medium effect size) which has been achieved in prior studies comparing different health care practices in Switzerland [33]. With a projected attrition rate of 33%, our targeted recruitment was 60 parents (control: n=30; intervention n=30) to reach a minimum sample size of 40 parents who completed the study (control: n=20; intervention: n=20), which provides 80% power using a 2-sided comparison of α .05 to detect a δ of 0.5.

### Statistical Analysis

For primary and secondary outcome measures, we used intention-to-treat analysis with repeated measures ANOVA to evaluate for between-group and within-group differences in the PACIC domain of goal-setting for intervention group versus control group while adjusting for fixed effects—timing of measurement (1: immediately after the visit and 2: 1 month after the visit) and intervention-by-time interaction—as well as random effects: provider cluster. We used a similar approach for our secondary outcomes. We conducted post hoc analyses of outcome variables with adjustment for population characteristics with statistically significant differences between subgroups. For baseline characteristics and feasibility outcome measures, we conducted descriptive analyses using Fisher exact test for binary and categorical variables and Wilcoxon sign-rank test for continuous variables. We used a statistical significance level of *P*<.05.

### Ethical Considerations

This study was approved by the Stanford Single Institutional Review Board (Protocol 32161, approved 12/22/2016) and registered on clinicaltrials.gov (NCT03620071). All participants gave documented informed consent prior to enrolling in the study. All potential participants had the option to opt out of the study if they were not interested. Provider participants were approached at routine division meetings when the study team presented the study, reviewed the consent form, and gave ample time for questions. Providers could provide consent during or after the team meetings. Potential parent participants were approached in-person in the waiting room for consent and enrollment prior to their clinic visit with a participating provider. We did not use remote recruitment due to concerns of intervention fidelity as GoalKeeper was designed to be used jointly with parents and providers during a clinic visit. Study team members presented the study, reviewed the consent form, and gave ample time for questions. Consent was obtained prior to the start of the clinic visit. Each participant was assigned a randomly generated 4-digit unique identifier. The key linking the unique identifier to participant name and contact information was stored in a separate database. Parent participants received gift card remuneration after surveys at t1 and t2 (US $10 each) and interviews at t3 (US $30). Providers received a US $5 gift card remuneration per completed survey and after interview completion totaling US $50 for the entire study period.

## Results

### Study Population

**Figure 2.**
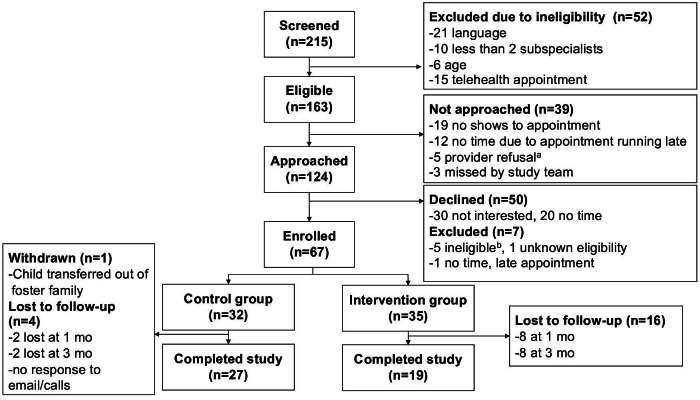
CONSORT (Consolidated Standards of Reporting Trials) flowchart. ^a^Provider rationale for refusal: parent ineligible due to underlying neurocognitive or behavioral health issue, under isolation protocol for suspected COVID-19, 1 emergent health care concern, visit changed to telehealth, patient late and had to see their non-primary provider instead. ^b^Participants ineligible at visit due to: no internet or email (1), language (1), legal (1), accompanied by non-guardian/non-primary caregiver (2).

We enrolled 67 parent-child dyads out of 124 eligible parents approached across 2 outpatient clinics and 11 medical providers. Of parent-child dyads, 32 were enrolled in the control group and 35 were enrolled in the intervention group. During the study period, 10 parent-child dyads (control: n=2; intervention: n=8) were lost to follow-up at T2, and 1 control group parent who was fostering a child with medical complexity was withdrawn due to the child being transferred out of their custody. We also had 2 providers lost to follow-up due to leaving the institution (CONSORT [Consolidated Standards of Reporting Trials] diagram in Figure 2).

Among parent participants, the mean age was 37.4 (SD 8.2) years, 50 (76%) were female participants, 32/66 (48%) identified as Caucasian, and 25 (38%) as Hispanic. Most were highly educated, with 51 (77%) having completed at least some college. Patients had a mean age of 5.6 (SD 0.5) years and 24 (36%) were female participants. All saw multiple subspecialties (median 3 [IQR 2‐4]). Most had technology dependence (34/66, 52%) and neurodevelopmental delay (36/66, 55%). Only the ventriculoperitoneal shunt was statistically significantly different between the control (0) and intervention (4) groups. The providers were mostly medical doctors (9/11, 82%), female participants (7/11, 64%), and neurologists (6/11, 54%). Parent, child, and provider characteristics are detailed in Table 1, Table S3 in Multimedia Appendix 1, and Table S4 in Multimedia Appendix 1, respectively.

**Table 1.** Population characteristics.

Characteristic	Total, n=66	Control, n=31	Intervention, n=35	*P* value^a^
Age, mean (SD)	37.4 (8.2)	36.4 (8.7)	38.3 (7.8)	.35
Sex, n (%)				.4
Female	50 (76)	22 (71)	28 (80)	
Race/ethnicity^b^, n (%)				.38
African American	2 (3)	0 (0)	2 (6)	
American Indian	2 (3)	1 (3)	1 (3)	
Asian	7 (11)	4 (13)	3 (9)	
Caucasian	32 (48)	15 (48)	17 (49)	
Hispanic	25 (38)	11 (35)	14 (40)	
Other	6 (9)	5 (16)	1 (3)	
Insurance, n (%)				.26
Public	30 (45)	11 (35)	19 (54)	
Private	31 (47)	13 (42)	18 (51)	
Other	20 (30)	13 (42)	7 (20)	
Don’t know	3 (5)	1 (3)	2 (6)	
Household size, mean (SD)	4.2 (1.3)	4.2 (1.6)	4.1 (1.0)	.76
Marital status, n (%)				.88
Single	17 (26)	7 (23)	10 (29)	
Married/living with partner	46 (69)	22 (71)	24 (69)	
Education level, n (%)				.63
Some high school	3 (2)	1 (3)	2 (6)	
High school diploma	10 (15)	6 (19)	4 (11)	
Some college	15 (23)	5 (16)	10 (29)	
College degree	22 (33)	10 (32)	12 (34)	
Advanced degree	14 (21)	7 (23)	7 (20)	

a Statistical significance level is *P*≤.05.

b Participants could select all categories that apply.

**Figure 3.**
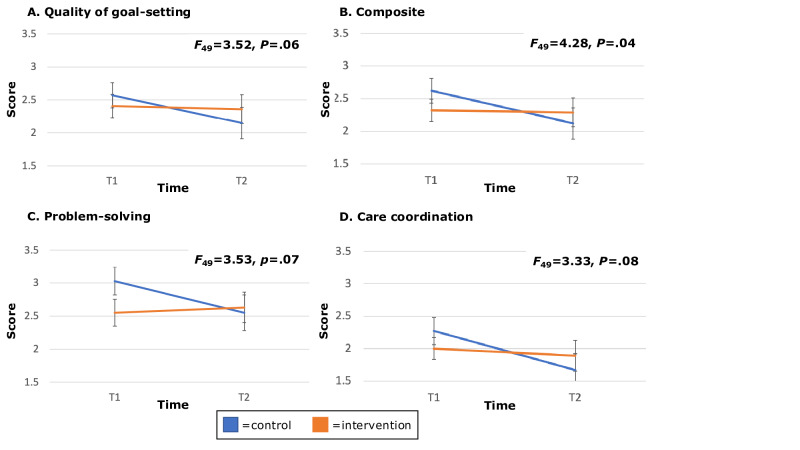
*F* reported is for intervention by time.

For our primary outcome measure, quality of goal-setting, scores were lower for the intervention group at t1 when compared to the control group, 2.41 (SD 1.07) versus 2.57 (SD 1.06). At 1-month follow-up (t2), the reverse was true with scores of 2.36 (SD 1.12) for the intervention group versus 2.15 (SD 1.23) for the control group. Quality of goal-setting between t1 and t2 remained stable for the intervention group but decreased for the control group (δ=0.03 vs −0.43, *F*_49_=3.52, *P*=.06). Similar patterns exist for other PACIC outcome measures of problem-solving, care coordination, and overall care quality (composite). For other secondary outcome measures, parenting distress at t1 was higher in the intervention group when compared to the control group (mean [SD] 27.9 [11.6] vs 22.2 [7.6]; *P*=.02). There were no significant group differences in parent report of quality of shared decision-making or quality of doctor-patient communication (Table 2 and Figure 3). For post hoc analyses of ventriculoperitoneal shunt, the sample size of 4 was too small for valid statistical analysis.

**Table 2.** Efficacy outcomes.

Outcome	T1		T2		*F* test (*df*)^a^	*P* value^b^
	Control, n=30	Intervention, n=35	Control, n=27	Intervention, n=27		
Quality of chronic illness care (PACIC^c^), mean (SD)						
Composite	2.62 (1.03)	2.32 (1.02)	2.12 (1.23)	2.29 (1.15)	4.28 (49)	.04
Goal-setting	2.57 (1.06)	2.41 (1.07)	2.15 (1.23)	2.36 (1.12)	3.52 (49)	.06
Problem-solving	3.03 (1.16)	2.55 (1.17)	2.55 (1.38)	2.63 (1.20)	3.53 (49)	.07
Coordination	2.27 (1.14)	2.00 (1.01)	1.67 (1.30)	1.89 (1.27)	3.33 (49)	.08
High-quality shared decision-making, n (%)	22 (73)	24 (69)	20 (74)	19 (70)	0.15 (49)	.70
Quality of communication (Communication Assessment Tool), mean (SD)	4.66 (0.78)	4.60 (0.52)	—^d^	—	—	.71
Parenting distress (Parenting Stress Index), mean (SD)	22.23 (7.66)	27.89 (11.61)	—	—	—	.02

a *F* reported is for intervention by time.

b Statistical significance level is *P*≤.05.

c PACIC: Patient Assessment of Care for Chronic Illness Care.

d not available.

### Intervention Fidelity

Among the intervention group, 34 of 35 parents successfully used GoalKeeper with their provider to set goals during the clinic visit. For 1 parent, the provider decided during the visit that it was not an appropriate time to use GoalKeeper, but to preserve an intention-to-treat approach, that parent remained in the intervention group to complete the survey measures. Parents and providers set a median of 2.5 (IQR 1.5-3) goals per initial clinic visit, and providers assigned a median of 3 (IQR 3-4) tracking templates to each parent. Each provider saw a median of 2.5 (IQR 2-5) parent participants . After the initial clinic visit, parents input information into GoalKeeper a median of 0 (IQR 0-3) times and providers viewed tracked data a median of 0.5 (IQR 0-1) times throughout the study period. A total of 19 parents (CPCC: n=8; neurology: n=11) did not enter any data into the tracking templates, while 13 parents (CPCC: n=4; neurology: n=9) entered data 1‐10 times, and 3 parents, all from CPCC, entered data over 10 times. In total, 87 goals were set with the most common domains being development (parents: n=10; unique goals: n=16), child nutrition (parents: n=13; unique goals: n=13), and seizure control (parents: n=10; unique goals: n=10). There were no statistically significant differences in participant and patient characteristics between those who used the tracking module more extensively than those who did not. Of the 19 parents who completed the exit survey, 13 (68%) parents would recommend GoalKeeper to other parents. A total of 6 (67%) providers would recommend GoalKeeper to other providers (Table S3 in Multimedia Appendix 1).

In exit interviews, participants identified facilitators to GoalKeeper use to be the shared nature of the goal-setting activity, the tool being accessible by the internet, and the ability to set long-term goals as facilitators to tool use. Per parent participants, the main barrier for sustained tool use after the initial goal-setting clinic visit was lack of integration in the EHR and patient portal. Additional details about the interview results and the tool’s feasibility and acceptability are reported in a companion manuscript [26].

## Discussion

### Principal Results

Our study demonstrated that the GoalKeeper intervention, a novel goal-setting platform for outpatient care of complex chronic illness, resulted in a better maintenance of overall chronic illness care quality, with no statistically significant impact on goal-setting, problem-solving, or coordination. Intervention fidelity results indicate that the sustained effect of GoalKeeper on care quality was likely the result of participant engagement with the intervention’s goal elicitation module, in contrast to its tracking module, which had very low participant engagement. Furthermore, we found that the intervention is both feasible and acceptable in the context of busy outpatient care, in both primary and subspecialty care settings. Together, these results suggest that technology supports for goal-setting may be an effective way to maintain high-quality care in between clinic visits for children with medical complexity, but GoalKeeper itself needs further refinement to improve user experience. However, given the nonstatistically significant findings in individual PACIC domains, the exact mechanism for this remains unclear.

The sustained quality of care for those in the intervention group is notable compared to the decline in quality of care for the control group, even though the initial quality of care scores (PACIC composite and domain) were lower for the intervention group than the controls. One potential explanation for these initial lower scores may be that the parents in the intervention group were more stressed as indicated by the parenting distress scores. Additionally, the COVID-19 pandemic may have contributed to greater parent stress in the intervention group, since the control group was enrolled earlier in the trial, although this was not a theme found in the exit interviews. Since we collected baseline data after the encounter, another explanation may be a learning curve that participants experienced due to changing the dynamic of a clinic visit through the use of a new tool (GoalKeeper), which may cause the visit to seem of poorer quality due to the challenge of learning a new approach to care [34]. A final possible explanation is simple random variation, which justifies our choice of a randomized controlled trial design to examine intervention effects. Based on our observed participant and patient characteristics, our randomization approach appears strong and thus makes it unlikely that care quality scores were lower due to higher medical complexity or disparities in care delivery.

### Comparison With Prior Work

Nonetheless, given the nonstatistically significant findings in individual PACIC domains, further research is necessary to extend and enhance the effectiveness of such platforms on care quality. Our results build on prior studies suggesting tools to improve care for children with medical complexity need to elicit and track family-centered goals [9,35]. Family engagement remains an urgent research priority for children with medical complexity, yet their providers report barriers including insufficient communication skills and supports [36,37]. Providers in our study acknowledged that they, too, struggled with the communication around goals with parents of children with medical complexity but endorsed that GoalKeeper facilitated better communication about parents’ goals. Multidisciplinary care coordination programs that included family-centered goal-setting found that family-centered goals helped programs emphasize family priorities [11]. However, many approaches to family-centered goal-setting have used resource-intensive approaches, which face barriers to implementation including the scalability and replicability of programs across multiple centers. Our intervention may enable these programs to extend their reach by acting as an adjunct intervention in clinical care whereby family-centered goal-setting can be achieved without additional extended team support. Future work on tracking and adapting the goals longitudinally could further sustain engagement and lead to evaluations of how long-term care is affected by family-centered goals.

While our study demonstrates high initial adoption of GoalKeeper based on goal elicitation module use, future studies should investigate the mechanisms of effect and tools to sustain engagement, such as the effects of a more sophisticated tracking module. Other digital health interventions have initial high levels of adoption that decrease dramatically over time [38]. Prior studies have demonstrated that self-monitoring interventions may result in sustained engagement even if the initial motivation for using the app was different [39]. In our study, the design of the tracking module was not the focus of intervention development. Since other existing tools to improve care for children with medical complexity focus on symptom tracking, we wanted to avoid duplicative efforts [40,41]. Future directions should explore whether integration of the Goal Elicitation module with existing symptom trackers will improve sustained use of GoalKeeper. Sustained use of digital health interventions after the initial adoption period has also been associated with user perceptions of usefulness, ease of use, fulfillment of user expectations, and satisfaction with the app [42]. Future evaluations should test whether these mechanisms can sustain use of our intervention in larger populations and in populations that prefer other languages.

Study findings also suggest additional design and implementation considerations for health-information technology platforms that aim to improve care for chronic conditions. Goal elicitation structures, similar to those developed for GoalKeeper, should be embedded across discrete elements of the EHR—especially the encounter note, the after-visit summary, and the patient portal. Additionally, goal-elicitation training should be integrated with provider-training modules, including simulated patient encounters, to enhance provider practice. While standalone tools could improve accessibility in community-based care, this study’s findings suggest high feasibility and acceptability for integrating adaptive, chronic-illness management technology into existing workflows.

### Limitations

Study findings should be interpreted in the context of a few limitations. This study was conducted in a single children’s health system, which may not be reflective of the practice at other institutions caring for children with medical complexity. However, the CPCC was part of a multicenter collaboration to develop care coordination programs for children with medical complexity, so some program similarities may exist across these collaborating sites. More participants in the intervention group were lost to follow-up, which may have introduced selection bias, with those with poorer experiences with the intervention or with higher medical needs choosing not to continue to participate in the study. However, there were no statistically significant differences in parent and child characteristics between those lost to follow-up and those who completed the study. Notably, participants had to have home internet access, and while the divide between internet access is narrowing, this could have excluded populations with lower household income [43]. The majority of our study participants also had at least some college education, which is reflective of the local population. Overall, our study period was quite short. Thus, the long-term intervention use, particularly on repeat clinic visits, was not observed. The effects of long-term intervention use should be assessed with future studies.

### Conclusions

Technologies that support real-time, shared goal-setting between a patient and their medical provider may serve as an adjunct to high-quality care of complex chronic illness. Informed by in-depth qualitative research and understanding of children with medical complexity-specific care needs and goals, health-technology tools like GoalKeeper are likely to be feasible and acceptable when integrated into both primary care and subspecialty care outpatient settings. Furthermore, in the context of learning health systems and emerging, generative artificial intelligence solutions, such platforms are likely to become easier to implement, scale, and study across care systems. As a result, goal-concordant care technologies, like Goalkeeper, co-designed with patients and caregivers in the context of a rigorous behavioral science framework, hold promise to improve care outcomes across a wide range of chronic and comorbid conditions.

## Supplementary material

10.2196/70402 Multimedia Appendix 1 Supplemental figures and tables.

10.2196/70402 Multimedia Appendix 2 Interview guides.

10.2196/70402 Checklist 1 CONSORT (Consolidated Standards of Reporting Trials)-EHEALTH checklist.

## Abbreviations:

CFIR: Consolidated Framework for Implementation Research
CONSORT: Consolidated Standards of Reporting Trials
CPCC: complex primary care clinics
EHR: electronic health record
PACIC: Patient Assessment of Care for Chronic Illness Care
REDCap: Research Electronic Data Capture
