# Supplementary material for: Novel Web-Based Technology to Promote Goal-Setting in Complex Chronic Illness: Randomized Controlled Trial
Source: JMIR Hum Factors. 2026 Feb 20;13:e70402. doi: 10.2196/70402 (PMC12923101; doi:10.2196/70402)
Supplement: Multimedia Appendix 1 [file humanfactors-v13-e70402-s001.docx]

**Figure S1. Recruitment period by clinic using stepped wedge design**

**Figure S2. Data collection time points**

**Table S1. Child characteristics**

| **Child Characteristics** | **Total (%) n= 66** | **Control n= 31** | **Intervention n=35** | **p-value^a^** |
| --- | --- | --- | --- | --- |
| **Age- mean (SD)** | 5.6 (0.5) | 5.3 (3.5) | 5.9 (3.8) | 0.54 |
| **Sex- Female** | 24 (36) | 8 (26) | 16 (46) | 0.1 |
| **Race/Ethnicity** |  |  |  | 0.73 |
| African American | 1 (2) | 0 (0) | 1 (3) |  |
| American Indian | 2 (3) | 1 (3) | 1 (3) |  |
| Asian | 9 (14) | 4 (13) | 5 (14) |  |
| Caucasian | 34 (52) | 16 (52) | 18 (51) |  |
| Hispanic | 29 (44) | 13 (42) | 16 (46) |  |
| Other | 7 (11) | 5 (16) | 2 (6) |  |
| **Children with Special Health Care Needs Screener questions** | | | |  |
| Needs or uses prescription medicines | 44 (67) | 26 (84) | 18 (51) | 0.23 |
| Needs or uses more medical care than usual | 51 (77) | 29 (94) | 22 (63) | 0.33 |
| Functional limitations more than usual | 47 (71) | 28 (90) | 19 (54) | 0.19 |
| Needs or uses special therapies | 54 (82) | 30 (97) | 24 (69) | 0.41 |
| Needs or uses treatment for emotional/developmental/behavioral issues | 30 (45) | 18 (58) | 12 (34) | 0.27 |
| **Subspecialists** |  |  |  |  |
| Cardiology | 19 (29) | 7 (23) | 12 (34) | 0.25 |
| Neurology | 50 (76) | 23 (74) | 27 (77) | 0.57 |
| Pulmonology | 30 (45) | 12 (39) | 18 (51) | 0.27 |
| Development | 35 (53) | 15 (48) | 20 (57) | 0.4 |
| Gastroenterology | 39 (59) | 17 (55) | 22 (63) | 0.32 |
| Occupational therapy | 45 (68) | 20 (65) | 25 (71) | 0.46 |
| Speech therapy | 33 (50) | 18 (58) | 15 (43) | 0.6 |
| Physical therapy | 44 (67) | 19 (61) | 25 (71) | 0.37 |
| Other | 24 (36) | 11 (35) | 13 (37) | 0.68 |
| **Technology dependence** |  |  |  |  |
| Ventriculoperitoneal shunt | 4 (6) | 0 (0) | 4 (11) | 0.05 |
| Gastrostomy tube | 22 (33) | 9 (29) | 13 (37) | 0.39 |
| Tracheostomy | 3 (5) | 1 (3) | 2 (6) | 0.56 |
| Other | 11 (17) | 5 (16) | 6 (17) | 0.76 |
| None | 32 (48) | 17 (55) | 15 (43) | 0.72 |
| **Neurodevelopmental delay** |  |  |  |  |
| Intellectual disability | 29 (44) | 11 (35) | 18 (51) | 0.19 |
| Cerebral palsy | 13 (20) | 5 (16) | 8 (23) | 0.41 |
| Visual impairment | 12 (18) | 5 (16) | 7 (20) | 0.56 |
| Hearing deficit | 6 (9) | 4 (13) | 2 (6) | 0.41 |
| None | 30 (45) | 15 (48) | 15 (43) | 1 |

1. Statistical significance level is p≤0.05.

**Table S2. Provider characteristics**

| **Characteristics (%)** | **Total** |
| --- | --- |
|  | **n=11** |
| **Sex- Female** | 7 (64) |
| **Specialty** |  |
| General pediatrics | 5 (46) |
| Child neurology | 6 (54) |
| **Degree** |  |
| MD | 9 (82) |
| DO | 1 (9) |
| NP | 1 (9) |

**Table S3. User survey results for parent and provider participants**

| **Parent survey questions (n=19)** | **Agree/**  **Strongly Agree**  **n (%)** | **Disagree/**  **Strongly disagree**  **n (%)** | **Undecided**  **n (%)** |  |
| --- | --- | --- | --- | --- |
| Overall, the GoalKeeper tool is useful. | 9 (47) | 2 (11) | 8 (42) |  |
| I would use the GoalKeeper tool in the future. | 11 (58) | 3 (16) | 5 (26) |  |
| I would recommend GoalKeeper to others | 13 (68) | 1 (5) | 5 (26) |  |
| GoalKeeper helped me think about the kinds of goals I would like to set for my child. | 15 (79) | 0 (0) | 4 (21) |  |
| GoalKeeper helped me and my child's doctor talk about goals for my child. | 10 (53) | 4 (21) | 5 (26) |  |
| It was easy to set up an account. | 9 (47) | 1 (5) | 9 (47) |  |
| It was easy to enter my goals. | 14 (74) | 2 (11) | 2 (11) |  |
| It was easy to track the goals I set with my child's doctor. | 12 (63) | 2 (11) | 4 (21) |  |
| **Provider survey questions (n=9)** | | | | |
| Overall, the GoalKeeper tool is useful. | 7 (78) | 1 (11) | 1 (11) |  |
| Using GoalKeeper helps me start important conversations with my patients' families | 6 (67) | 1 (11) | 1 (11) |  |
| Using GoalKeeper helps me start important conversations about the future with my patients' families | 7 (78) | 1 (11) | 1 (11) |  |
| Using GoalKeeper fits within my typical workflow | 3 (33) | 6 (67) |  |  |
| Using GoalKeeper helps me and my patients' families to set actionable goals | 6 (67) | 1 (11) | 2 (22) |  |
| I found the example goals in GoalKeeper to be helpful. | 8 (89) | 0 (0) | 1 (11) |  |
| It was easy to enter my patient's goals in GoalKeeper. | 8 (89) | 1 (11) | 1 (11) |  |
| It was easy to track my patient's goals in GoalKeeper. | 7 (78) | 1 (11) | 1 (11) |  |
| Would you recommend GoalKeeper to others? (yes/no) | 6 (67) |  |  |  |
