# Supplementary material for: Novel Web-Based Technology to Promote Goal-Setting in Complex Chronic Illness: Randomized Controlled Trial
Source: JMIR Hum Factors. 2026 Feb 20;13:e70402. doi: 10.2196/70402 (PMC12923101; doi:10.2196/70402)
Supplement: Multimedia Appendix 2 [file humanfactors-v13-e70402-s002.docx]

**GK Intervention Group Family Exit Interview**

***Preamble:* Thank you for participating in our study. You are the expert for your child and your child’s care. In order to better understand your experiences with the GoalKeeeper intervention, we would like to ask you some additional questions. This information will help our team better understand your experiences and help use develop future versions of the GoalKeeper intervention, which we will refer to as GoalKeeper from now on. There are no right or wrong answers to these questions. Please feel free to be as open and honest in your answers so that we can better understand how to support families like you and your child.**

**Intervention AND individuals AND tension for change AND knowledge and beliefs (about the intervention):**

1. Tell me about your experience with GoalKeeper.
   1. How did you use GoalKeeper?
   2. When was using GoalKeeper helpful?
   3. When was using GoalKeeper not helpful?
2. How did GoalKeeper affect what you did on a daily basis?

**Intervention AND inner setting AND patient needs and resources/tension for change/relative advantage:**

1. How does GoalKeeper compare to other tools you use to manage your child’s care?
   1. What advantages/ disadvantages does GoalKeeper have?

**Intervention AND individuals AND relative advantage:**

1. What was made more difficult with GoalKeeper?
2. What was made easier with GoalKeeper?

**Intervention AND complexity:**

1. What made it hard for you to use GoalKeeper?
2. What made it easier for you to use GoalKeeper?

**Intervention AND adaptability:**

1. What would make GoalKeeper more useful?
2. What components should be left unchanged?

**Process AND champions:**

1. Of the people you know, who are likely to champion the intervention?
2. Of the people you look to for advice, who would likely use GoalKeeper?
3. Of your child’s care team members both medical and nonmedical, who would you want to use GoalKeeper with?

**Inner setting AND compatibility AND individuals:**

1. How well does Goalkeeper fit with your needs as your child’s caregiver?
2. How well does Goalkeeper fit with your child’s care needs?
3. How well does GoalKeeper fit with the needs of your child’s medical providers?

**Intervention AND relative advantage:**

1. Is there a strong need for GoalKeeper?
   1. Why or why not?
   2. Do you think other parents like you need this intervention? Why or why not?

**GoalKeeper unique questions: (10 min)**

**Intervention AND individuals AND compatibility:**

1. Was there anything in particular that you put into GoalKeeper that you wanted your provider to look at?

**Individual state of change:**

1. Since using GoalKeeper, tell me about your experiences working with your child’s doctor to set goals for your child?
   - Since using GoalKeeper, how has the process of setting goals changed?
   - Since using GoalKeeper, to what extent do you feel like you and your child’s doctor work together to set goals for your child?
   - How has GoalKeeper helped you remember the goals that you and your child’s doctor set?
   - How has GoalKeeper helped you track progress on the goals that you and your child’s doctor set?
   - Since using GoalKeeper, how does having set goals affect your other activities?
   - Since using GoalKeeper, has goal-setting helped to organize care activities?
   - Did you use GoalKeeper with other providers?
     1. How did you use it?
     2. How was it helpful?

**Logged data: (10 min)**

Now I’m going to ask you more specific questions about how you used GoalKeeper. For this part, I’m going to log onto GoalKeeper in case we need to refer to them. I can log into a test patient unless you prefer to log into your account. (Log into GK.)

1. What parts of GoalKeeper did you use?
2. What parts of GoalKeeper did you not use?
3. Use user information to dig deeper into what they did use and why + what they didn’t use and why.

**Keep using:**

1. Would you want to keep using GoalKeeper?

**Active:**

1. Now that the study is over, would you like us to keep your account open? You will see the platform change periodically but can continue to log information in the system.

**Misc:**

1. Those are all the questions we have for your today. What other comments, questions, or suggestions do you have?

**COVID:**

What are the most challenging parts of caring for your child during the coronavirus pandemic?

                How are you dealing with these challenges?

**RCT Exit Interview Questions for Providers**

***Preamble:* Thank you for participating in our study. To better understand your experiences with the GoalKeeeper intervention, we would like to ask you some additional questions. This information will help our team better understand your experiences and help use develop future versions of the GoalKeeper intervention, which we will refer to as GoalKeeper from now on. There are no right or wrong answers to these questions. Please feel free to be as open and honest in your answers so that we can better understand how to support providers like you and your patients’ families.**

**Inner setting AND tension for change AND knowledge and beliefs about the intervention:**

1. Tell me about your experience with GoalKeeper.
   1. How did you use GoalKeeper?
   2. When was using GoalKeeper helpful?
   3. When was using GoalKeeper not helpful?

**Intervention AND relative advantage:**

1. What were some of the challenges with using GoalKeeper?
   1. What was made more difficult with GoalKeeper?
   2. What was made easier with GoalKeeper?

**Intervention AND complexity:**

- 1. What made it hard for you to use GoalKeeper?
  2. What made it easier for you to use GoalKeeper?

**Intervention AND adaptability:**

1. What would have made GoalKeeper more useful?
2. What components should be left unchanged?

**GoalKeeper AND intervention AND individuals AND compatibility AND knowledge and beliefs AND individual state of change:**

1. Since using GoalKeeper, tell me about your experiences working with your patients’ families to set goals?
   1. Since using GoalKeeper, to what extent do you feel like you and your patients’ families work together to set goals? Does this happen with patients who did not have GoalKeeper?
   2. How has GoalKeeper helped you track progress on the goals that you and your patients’ families set?

**Logged data:**

1. What parts of GoalKeeper did you use?
2. What parts of GoalKeeper did you not use?
3. Use user information to dig deeper into what they did use and why + what they didn’t use and why.

**Keep using:**

1. Would you want to keep using GoalKeeper?

**Misc:**

1. Those are all the questions we have for your today. What other comments, questions, or suggestions do you have?

**COVID:**

What unique challenges did you face in caring for children with medical complexity during the COVID-19 pandemic?

What solutions did you come up with to address these challenges?
