# Supplementary material for: Novel Web-Based Technology to Promote Goal-Setting in Complex Chronic Illness: Randomized Controlled Trial
Source: JMIR Hum Factors. 2026 Feb 20;13:e70402. doi: 10.2196/70402 (PMC12923101; doi:10.2196/70402)
Supplement: Checklist 1 [file humanfactors-v13-e70402-s003.pdf]

CONSORT-EHEALTH (V 1.6.1) - Submission/Publication Form

The CONSORT-EHEALTH checklist is intended for authors of randomized trials evaluating web-based and Internet-based applications/interventions, including mobile interventions, electronic games (incl multiplayer games), social media, certain telehealth applications, and other interactive and/or networked electronic applications. Some of the items (e.g. all subitems under item 5 - description of the intervention) may also be applicable for other study designs.

The goal of the CONSORT EHEALTH checklist and guideline is to be
a) a guide for reporting for authors of RCTs,
b) to form a basis for appraisal of an ehealth trial (in terms of validity)

CONSORT-EHEALTH items/subitems are MANDATORY reporting items for studies published in the Journal of Medical Internet Research and other journals / scientific societies endorsing the checklist.

Items numbered 1., 2., 3., 4a., 4b etc are original CONSORT or CONSORT-NPT (non-pharmacologic treatment) items.
Items with Roman numerals (i., ii, iii, iv etc.) are CONSORT-EHEALTH extensions/ clarifications.

As the CONSORT-EHEALTH checklist is still considered in a formative stage, we would ask that you also RATE ON A SCALE OF 1-5 how important/useful you feel each item is FOR THE PURPOSE OF THE CHECKLIST and reporting guideline (optional).

Mandatory reporting items are marked with a red *.
In the textboxes, either copy & paste the relevant sections from your manuscript into this form - please include any quotes from your manuscript in QUOTATION MARKS,
or answer directly by providing additional information not in the manuscript, or elaborating on why the item was not relevant for this study.

YOUR ANSWERS WILL BE PUBLISHED AS A SUPPLEMENTARY FILE TO YOUR PUBLICATION IN JMIR AND ARE CONSIDERED PART OF YOUR PUBLICATION (IF ACCEPTED).
Please fill in these questions diligently. Information will not be copyedited, so please use proper spelling and grammar, use correct capitalization, and avoid abbreviations.

DO NOT FORGET TO SAVE AS PDF _AND_ CLICK THE SUBMIT BUTTON SO YOUR ANSWERS ARE IN OUR DATABASE !!!

Citation Suggestion (if you append the pdf as Appendix we suggest to cite this paper in the caption):
Eysenbach G, CONSORT-EHEALTH Group
CONSORT-EHEALTH: Improving and Standardizing Evaluation Reports of Web-based and Mobile Health Interventions
J Med Internet Res 2011;13(4):e126
URL: http://www.jmir.org/2011/4/e126/
doi: 10.2196/jmir.1923
PMID: 22209829

jody.lin@gmail.com Switch account

Draft not saved

Not shared

* Indicates required question

Unable to save your draft

Your name *

First Last

Jody Lin

Primary Affiliation (short), City, Country *

University of Toronto, Toronto, Canada

University of Utah

Your e-mail address *

abc@gmail.com

jody.lin@hsc.utah.edu

Title of your manuscript *

Provide the (draft) title of your manuscript.

Novel web-based technology to promote goal setting in complex chronic illness: a randomized controlled trial

Name of your App/Software/Intervention *

If there is a short and a long/alternate name, write the short name first and add the long name in brackets.

GoalKeeper

Evaluated Version (if any)

e.g. "V1", "Release 2017-03-01", "Version 2.0.27913"

Your answer

Language(s) *

What language is the intervention/app in? If multiple languages are available, separate by comma (e.g. "English, French")

English

Unable to save your draft

URL of your Intervention Website or App

e.g. a direct link to the mobile app on app in appstore (itunes, Google Play), or URL of the website. If the intervention is a DVD or hardware, you can also link to an Amazon page.

Your answer

URL of an image/screenshot (optional)

Your answer

Accessibility *

Can an enduser access the intervention presently?

- ○ access is free and open
- ○ access only for special usergroups, not open
- ○ access is open to everyone, but requires payment/subscription/in-app purchases
- ◉ app/intervention no longer accessible
- ○ Other:

Primary Medical Indication/Disease/Condition *

e.g. "Stress", "Diabetes", or define the target group in brackets after the condition, e.g. "Autism (Parents of children with)", "Alzheimers (Informal Caregivers of)"

Medical Complexity (Parents of child with)

Primary Outcomes measured in trial *

comma-separated list of primary outcomes reported in the trial

difference in parent-reported quality of goal-se

Secondary/other outcomes

Are there any other outcomes the intervention is expected to affect?

care quality, quality of shared decision making, quality of parent-provider communication, and parenting stress

Unable to save your draft

Recommended "Dose" *

What do the instructions for users say on how often the app should be used?

- ○ Approximately Daily
- ◉ Approximately Weekly
- ○ Approximately Monthly
- ○ Approximately Yearly
- ○ "as needed"
- ○ Other:

Approx. Percentage of Users (starters) still using the app as recommended after 3 months *

- ◉ unknown / not evaluated
- ○ 0-10%
- ○ 11-20%
- ○ 21-30%
- ○ 31-40%
- ○ 41-50%
- ○ 51-60%
- ○ 61-70%
- ○ 71%-80%
- ○ 81-90%
- ○ 91-100%
- ○ Other:

Unable to save your draft

Overall, was the app/intervention effective? *

- ( ) yes: all primary outcomes were significantly better in intervention group vs control
- ( ) partly: SOME primary outcomes were significantly better in intervention group vs control
- (•) no statistically significant difference between control and intervention
- ( ) potentially harmful: control was significantly better than intervention in one or more outcomes
- ( ) inconclusive: more research is needed
- ( ) Other:

Article Preparation Status/Stage *

At which stage in your article preparation are you currently (at the time you fill in this form)

- ( ) not submitted yet - in early draft status
- ( ) not submitted yet - in late draft status, just before submission
- ( ) submitted to a journal but not reviewed yet
- (•) submitted to a journal and after receiving initial reviewer comments
- ( ) submitted to a journal and accepted, but not published yet
- ( ) published
- ( ) Other:

Journal *

If you already know where you will submit this paper (or if it is already submitted), please provide the journal name (if it is not JMIR, provide the journal name under "other")

- ( ) not submitted yet / unclear where I will submit this
- (•) Journal of Medical Internet Research (JMIR)
- ( ) JMIR mHealth and UHealth
- ( ) JMIR Serious Games
- ( ) JMIR Mental Health
- ( ) JMIR Public Health
- ( ) JMIR Formative Research
- ( ) Other JMIR sister journal
- ( ) Other:

Unable to save your draft

Is this a full powered effectiveness trial or a pilot/feasibility trial? *

- [x] Pilot/feasibility
- [ ] Fully powered

Manuscript tracking number *

If this is a JMIR submission, please provide the manuscript tracking number under "other" (The ms tracking number can be found in the submission acknowledgement email, or when you login as author in JMIR. If the paper is already published in JMIR, then the ms tracking number is the four-digit number at the end of the DOI, to be found at the bottom of each published article in JMIR)

- [ ] no ms number (yet) / not (yet) submitted to / published in JMIR
- [x] Other: 70402

TITLE AND ABSTRACT

1a) TITLE: Identification as a randomized trial in the title

1a) Does your paper address CONSORT item 1a? *

I.e does the title contain the phrase "Randomized Controlled Trial"? (if not, explain the reason under "other")

- [x] yes
- [ ] Other:

1a-i) Identify the mode of delivery in the title

Identify the mode of delivery. Preferably use “web-based” and/or “mobile” and/or “electronic game” in the title. Avoid ambiguous terms like “online”, “virtual”, “interactive”. Use “Internet-based” only if Intervention includes non-web-based Internet components (e.g. email), use “computer-based” or “electronic” only if offline products are used. Use “virtual” only in the context of “virtual reality” (3-D worlds). Use “online” only in the context of “online support groups”. Complement or substitute product names with broader terms for the class of products (such as “mobile” or “smart phone” instead of “iphone”), especially if the application runs on different platforms.

	1	2	3	4	5	
subitem not at all important	○	○	●	○	○	essential

Clear selection

Unable to save your draft

Does your paper address subitem 1a-i? *

Copy and paste relevant sections from manuscript title (include quotes in quotation marks "like this" to indicate direct quotes from your manuscript), or elaborate on this item by providing additional information not in the ms, or briefly explain why the item is not applicable/relevant for your study

We use the term "web-based technology" in our title

1a-ii) Non-web-based components or important co-interventions in title

Mention non-web-based components or important co-interventions in title, if any (e.g., “with telephone support”).

	1	2	3	4	5	
subitem not at all important	(●)	( )	( )	( )	( )	essential

Clear selection

Does your paper address subitem 1a-ii?

Copy and paste relevant sections from manuscript title (include quotes in quotation marks "like this" to indicate direct quotes from your manuscript), or elaborate on this item by providing additional information not in the ms, or briefly explain why the item is not applicable/relevant for your study

There were no non-web-based components of the intervention.

1a-iii) Primary condition or target group in the title

Mention primary condition or target group in the title, if any (e.g., “for children with Type I Diabetes”) Example: A Web-based and Mobile Intervention with Telephone Support for Children with Type I Diabetes: Randomized Controlled Trial

	1	2	3	4	5	
subitem not at all important	( )	( )	(●)	( )	( )	essential

Clear selection

Does your paper address subitem 1a-iii? *

Copy and paste relevant sections from manuscript title (include quotes in quotation marks "like this" to indicate direct quotes from your manuscript), or elaborate on this item by providing additional information not in the ms, or briefly explain why the item is not applicable/relevant for your study

We use the term "complex chronic illness" in our title

Unable to save your draft

1b) ABSTRACT: Structured summary of trial design, methods, results, and conclusions

NPT extension: Description of experimental treatment, comparator, care providers, centers, and blinding status.

1b-i) Key features/functionalities/components of the intervention and comparator in the METHODS section of the ABSTRACT

Mention key features/functionalities/components of the intervention and comparator in the abstract. If possible, also mention theories and principles used for designing the site. Keep in mind the needs of systematic reviewers and indexers by including important synonyms. (Note: Only report in the abstract what the main paper is reporting. If this information is missing from the main body of text, consider adding it)

	1	2	3	4	5	
subitem not at all important	○	○	○	○	◉	essential

Clear selection

Does your paper address subitem 1b-i? *

Copy and paste relevant sections from the manuscript abstract (include quotes in quotation marks "like this" to indicate direct quotes from your manuscript), or elaborate on this item by providing additional information not in the ms, or briefly explain why the item is not applicable/relevant for your study

We describe the intervention in our abstract like this, "Intervention: GoalKeeper was co-designed by parents, providers, and computer scientists to include two modules: 1) Goal elicitation, used during a clinic visit. 2) Tracking, used between visits."

1b-ii) Level of human involvement in the METHODS section of the ABSTRACT

Clarify the level of human involvement in the abstract, e.g., use phrases like “fully automated” vs. “therapist/nurse/care provider/physician-assisted” (mention number and expertise of providers involved, if any). (Note: Only report in the abstract what the main paper is reporting. If this information is missing from the main body of text, consider adding it)

	1	2	3	4	5	
subitem not at all important	○	○	○	○	○	essential

Unable to save your draft

Does your paper address subitem 1b-ii?

Copy and paste relevant sections from the manuscript abstract (include quotes in quotation marks "like this" to indicate direct quotes from your manuscript), or elaborate on this item by providing additional information not in the ms, or briefly explain why the item is not applicable/relevant for your study

We clarified in the abstract, "by parents and providers of CMC"

1b-iii) Open vs. closed, web-based (self-assessment) vs. face-to-face assessments in the METHODS section of the ABSTRACT

Mention how participants were recruited (online vs. offline), e.g., from an open access website or from a clinic or a closed online user group (closed usergroup trial), and clarify if this was a purely web-based trial, or there were face-to-face components (as part of the intervention or for assessment). Clearly say if outcomes were self-assessed through questionnaires (as common in web-based trials). Note: In traditional offline trials, an open trial (open-label trial) is a type of clinical trial in which both the researchers and participants know which treatment is being administered. To avoid confusion, use “blinded” or “unblinded” to indicated the level of blinding instead of “open”, as “open” in web-based trials usually refers to “open access” (i.e. participants can self-enrol). (Note: Only report in the abstract what the main paper is reporting. If this information is missing from the main body of text, consider adding it)

	1	2	3	4	5	
subitem not at all important	○	○	○	○	○	essential

Does your paper address subitem 1b-iii?

Copy and paste relevant sections from the manuscript abstract (include quotes in quotation marks "like this" to indicate direct quotes from your manuscript), or elaborate on this item by providing additional information not in the ms, or briefly explain why the item is not applicable/relevant for your study

We used self-assessment via surveys outcomes as described, "Quality of goal-setting assessed by the Patient Assessment of Care for Chronic Illness Care (PACIC) goal-setting domain at baseline (t1) and 1 month (t2)."

1b-iv) RESULTS section in abstract must contain use data

Report number of participants enrolled/assessed in each group, the use/uptake of the intervention (e.g., attrition/adherence metrics, use over time, number of logins etc.), in addition to primary/secondary outcomes. (Note: Only report in the abstract what the main paper is reporting. If this information is missing from the main body of text, consider adding it)

	1	2	3	4	5	
subitem not at all important	○	○	○	○	○	essential

Unable to save your draft

Does your paper address subitem 1b-iv?

Copy and paste relevant sections from the manuscript abstract (include quotes in quotation marks "like this" to indicate direct quotes from your manuscript), or elaborate on this item by providing additional information not in the ms, or briefly explain why the item is not applicable/relevant for your study

We have added the following, "34 of 35 intervention parents successfully used GoalKeeper. "

1b-v) CONCLUSIONS/DISCUSSION in abstract for negative trials

Conclusions/Discussions in abstract for negative trials: Discuss the primary outcome - if the trial is negative (primary outcome not changed), and the intervention was not used, discuss whether negative results are attributable to lack of uptake and discuss reasons. (Note: Only report in the abstract what the main paper is reporting. If this information is missing from the main body of text, consider adding it)

	1	2	3	4	5	
subitem not at all important	○	○	○	○	○	essential

Does your paper address subitem 1b-v?

Copy and paste relevant sections from the manuscript abstract (include quotes in quotation marks "like this" to indicate direct quotes from your manuscript), or elaborate on this item by providing additional information not in the ms, or briefly explain why the item is not applicable/relevant for your study

Although our primary outcome was not statistically significant, we found statistical significance in care quality. Thus, our conclusion reads, "Technologies that support goal-setting during clinical encounters may augment health-system efforts to advance high-quality chronic illness care."

INTRODUCTION

2a) In INTRODUCTION: Scientific background and explanation of rationale

2a-i) Problem and the type of system/solution

Describe the problem and the type of system/solution that is object of the study: intended as stand-alone intervention vs. incorporated in broader health care program? Intended for a particular patient population? Goals of the intervention, e.g., being more cost-effective to other interventions, replace or complement other solutions? (Note: Details about the intervention are provided in “Methods” under 5)

	1	2	3	4	5	
subitem not at all	○	○	○	○	○	essential

Unable to save your draft

Does your paper address subitem 2a-i? *

Copy and paste relevant sections from the manuscript (include quotes in quotation marks "like this" to indicate direct quotes from your manuscript), or elaborate on this item by providing additional information not in the ms, or briefly explain why the item is not applicable/relevant for your study

The objective for this study was to "evaluate the effectiveness of a technology-based tool for eliciting and tracking shared goals in the ambulatory care of CMC."

2a-ii) Scientific background, rationale: What is known about the (type of) system

Scientific background, rationale: What is known about the (type of) system that is the object of the study (be sure to discuss the use of similar systems for other conditions/ diagnoses, if appropiate), motivation for the study, i.e. what are the reasons for and what is the context for this specific study, from which stakeholder viewpoint is the study performed, potential impact of findings [2]. Briefly justify the choice of the comparator.

	1	2	3	4	5	
subitem not at all important	○	○	○	○	○	essential

Does your paper address subitem 2a-ii? *

Copy and paste relevant sections from the manuscript (include quotes in quotation marks "like this" to indicate direct quotes from your manuscript), or elaborate on this item by providing additional information not in the ms, or briefly explain why the item is not applicable/relevant for your study

We identified the following gap: "Prior work suggests that shared care plans for CMC require common goals to be set synchronously by the provider and caregiver, then tracked asynchronously by both but has not previously been tested.[15] It is unknown whether technology-based tools can be effective in improving the care of CMC."

2b) In INTRODUCTION: Specific objectives or hypotheses

Does your paper address CONSORT subitem 2b? *

Copy and paste relevant sections from the manuscript (include quotes in quotation marks "like this" to indicate direct quotes from your manuscript), or elaborate on this item by providing additional information not in the ms, or briefly explain why the item is not applicable/relevant for your study

"We hypothesized that parents of CMC exposed to GoalKeeper would report higher care quality and higher quality goal-setting when compared to parents who received usual care."

METHODS

Unable to save your draft

3a) Description of trial design (such as parallel, factorial) including allocation ratio

Does your paper address CONSORT subitem 3a? *

Copy and paste relevant sections from the manuscript (include quotes in quotation marks "like this" to indicate direct quotes from your manuscript), or elaborate on this item by providing additional information not in the ms, or briefly explain why the item is not applicable/relevant for your study

"We conducted a prospective, stepped-wedge, randomized controlled trial of intervention efficacy."

3b) Important changes to methods after trial commencement (such as eligibility criteria), with reasons

Does your paper address CONSORT subitem 3b? *

Copy and paste relevant sections from the manuscript (include quotes in quotation marks "like this" to indicate direct quotes from your manuscript), or elaborate on this item by providing additional information not in the ms, or briefly explain why the item is not applicable/relevant for your study

No changes to methods occurred after trial commencement.

3b-i) Bug fixes, Downtimes, Content Changes

Bug fixes, Downtimes, Content Changes: ehealth systems are often dynamic systems. A description of changes to methods therefore also includes important changes made on the intervention or comparator during the trial (e.g., major bug fixes or changes in the functionality or content) (5-iii) and other “unexpected events” that may have influenced study design such as staff changes, system failures/downtimes, etc. [2].

	1	2	3	4	5	
subitem not at all important	○	○	○	○	○	essential

Does your paper address subitem 3b-i?

Copy and paste relevant sections from the manuscript (include quotes in quotation marks "like this" to indicate direct quotes from your manuscript), or elaborate on this item by providing additional information not in the ms, or briefly explain why the item is not applicable/relevant for your study

We did not have any unexpected events to the intervention.

Unable to save your draft

Does your paper address CONSORT subitem 4a? *

Copy and paste relevant sections from the manuscript (include quotes in quotation marks "like this" to indicate direct quotes from your manuscript), or elaborate on this item by providing additional information not in the ms, or briefly explain why the item is not applicable/relevant for your study

"All providers that were not trainees at these clinics were eligible for recruitment. We recruited a convenience sample of primary caregivers (e.g. parents) of the patients seen by enrolled providers in-person at the time of their clinic visit. We capped enrollment per provider cluster at 10 parents. Parents were eligible if they were ≥18 years, English-speaking, with a child with medical complexity <12 years. We excluded parents without home Internet access or with a known neurocognitive or psychiatric issues that precluded ability to offer informed consent. We excluded older children, who may have the capacity to participate in decision-making, since the intervention was not designed for interaction with children.[19] We defined medical complexity as meeting each of two criteria in the 12 months prior to study enrollment: 1) ambulatory visits with at least two different subspecialties affiliated with the medical center and 2) medical fragility due to technology dependence (gastrostomy tube, nasogastric tube, ventriculoperitoneal shunt, or tracheostomy) or clinic visits at CPCC.
"

4a-i) Computer / Internet literacy

Computer / Internet literacy is often an implicit “de facto” eligibility criterion - this should be explicitly clarified.

	1	2	3	4	5	
subitem not at all important	○	○	○	○	○	essential

Does your paper address subitem 4a-i?

Copy and paste relevant sections from the manuscript (include quotes in quotation marks "like this" to indicate direct quotes from your manuscript), or elaborate on this item by providing additional information not in the ms, or briefly explain why the item is not applicable/relevant for your study

"We recruited medical providers (MD, DO, NP, PA) from two ambulatory clinics that see a high proportion of CMC: pediatric neurology and complex primary care clinics (CPCC) at the main location of a single academic health center. The health center is part of a standalone tertiary children’s health system in northern California. All providers that were not trainees at these clinics were eligible for recruitment. We recruited a convenience sample of primary caregivers (e.g. parents) of the patients seen by enrolled providers in-person at the time of their clinic visit. We capped enrollment per provider cluster at 10 parents. Parents were eligible if they were ≥18 years, English-speaking, with a child with medical complexity <12 years. We excluded parents without home Internet access or with a known neurocognitive or psychiatric issues that precluded ability to offer informed consent. We excluded older children, who may have the capacity to participate in decision-making, since the intervention was not designed for interaction with children.[19] We defined medical complexity as meeting each of two criteria in the 12 months prior to study enrollment: 1) ambulatory visits with at least two different subspecialties affiliated with the medical center and 2) medical fragility due to technology dependence (gastrostomy tube, nasogastric tube, ventriculoperitoneal shunt, or tracheostomy) or clinic visits at CPCC."

Unable to save your draft

4a-ii) Open vs. closed, web-based vs. face-to-face assessments:

Open vs. closed, web-based vs. face-to-face assessments: Mention how participants were recruited (online vs. offline), e.g., from an open access website or from a clinic, and clarify if this was a purely web-based trial, or there were face-to-face components (as part of the intervention or for assessment), i.e., to what degree got the study team to know the participant. In online-only trials, clarify if participants were quasi-anonymous and whether having multiple identities was possible or whether technical or logistical measures (e.g., cookies, email confirmation, phone calls) were used to detect/prevent these.

	1	2	3	4	5	
subitem not at all important	○	○	○	○	○	essential

Does your paper address subitem 4a-ii? *

Copy and paste relevant sections from the manuscript (include quotes in quotation marks "like this" to indicate direct quotes from your manuscript), or elaborate on this item by providing additional information not in the ms, or briefly explain why the item is not applicable/relevant for your study

"We recruited a convenience sample of primary caregivers (e.g. parents) of the patients seen by enrolled providers in-person at the time of their clinic visit. "

4a-iii) Information giving during recruitment

Information given during recruitment. Specify how participants were briefed for recruitment and in the informed consent procedures (e.g., publish the informed consent documentation as appendix, see also item X26), as this information may have an effect on user self-selection, user expectation and may also bias results.

	1	2	3	4	5	
subitem not at all important	○	○	○	○	○	essential

Does your paper address subitem 4a-iii?

Copy and paste relevant sections from the manuscript (include quotes in quotation marks "like this" to indicate direct quotes from your manuscript), or elaborate on this item by providing additional information not in the ms, or briefly explain why the item is not applicable/relevant for your study

"Provider participants were approached at routine division meetings when the study team presented the study, reviewed the consent form, and gave ample time for questions. Providers could provide consent during or after the team meetings. Potential parent participants were approached in-person in the waiting room for consent and enrollment prior to their clinic visit with a participating provider. We did not use remote recruitment due to concerns of intervention fidelity as GoalKeeper was designed to be used jointly with parents and providers during a clinic visit. Study team members presented the study, reviewed the consent form, and gave ample time for questions. Consent was obtained prior to the start of the clinic visit. "

Unable to save your draft

4b) Settings and locations where the data were collected

Does your paper address CONSORT subitem 4b? *

Copy and paste relevant sections from the manuscript (include quotes in quotation marks "like this" to indicate direct quotes from your manuscript), or elaborate on this item by providing additional information not in the ms, or briefly explain why the item is not applicable/relevant for your study

"We recruited medical providers (MD, DO, NP, PA) from two ambulatory clinics that see a high proportion of CMC: pediatric neurology and complex primary care clinics (CPCC) at the main location of a single academic health center."

4b-i) Report if outcomes were (self-)assessed through online questionnaires

Clearly report if outcomes were (self-)assessed through online questionnaires (as common in web-based trials) or otherwise.

	1	2	3	4	5	
subitem not at all important	○	○	○	○	○	essential

Does your paper address subitem 4b-i? *

Copy and paste relevant sections from the manuscript (include quotes in quotation marks "like this" to indicate direct quotes from your manuscript), or elaborate on this item by providing additional information not in the ms, or briefly explain why the item is not applicable/relevant for your study

"Measures of primary and secondary outcomes were collected electronically immediately after the initial clinical encounter for both parents and providers following study enrollment (T1) and at 1-month post-encounter (T2). Additional implementation outcome survey measures and exit interviews were collected at 3-months post-encounter (T3) for parents and, for providers, at the end of study period for their last enrolled parent. (See Supplemental Figure 3.) All measures were collected via secure electronic survey tools and stored electronically via the Stanford REDCap. "

4b-ii) Report how institutional affiliations are displayed

Report how institutional affiliations are displayed to potential participants [on ehealth media], as affiliations with prestigious hospitals or universities may affect volunteer rates, use, and reactions with regards to an intervention.(Not a required item – describe only if this may bias results)

	1	2	3	4	5	
subitem not at all important	○	○	○	○	○	essential

Unable to save your draft

Does your paper address subitem 4b-ii?

Copy and paste relevant sections from the manuscript (include quotes in quotation marks "like this" to indicate direct quotes from your manuscript), or elaborate on this item by providing additional information not in the ms, or briefly explain why the item is not applicable/relevant for your study

All participants were providers or parents of children seen at the academic center where the study was conducted. The consent form and study team members identified themselves as affiliates of the academic center.

5) The interventions for each group with sufficient details to allow replication, including how and when they were actually administered

5-i) Mention names, credential, affiliations of the developers, sponsors, and owners

Mention names, credential, affiliations of the developers, sponsors, and owners [6] (if authors/evaluators are owners or developer of the software, this needs to be declared in a “Conflict of interest” section or mentioned elsewhere in the manuscript).

	1	2	3	4	5	
subitem not at all important	○	○	○	○	○	essential

Does your paper address subitem 5-i?

Copy and paste relevant sections from the manuscript (include quotes in quotation marks "like this" to indicate direct quotes from your manuscript), or elaborate on this item by providing additional information not in the ms, or briefly explain why the item is not applicable/relevant for your study

There are no affiliated developers, sponsors, and owners for the intervention.

5-ii) Describe the history/development process

Describe the history/development process of the application and previous formative evaluations (e.g., focus groups, usability testing), as these will have an impact on adoption/use rates and help with interpreting results.

	1	2	3	4	5	
subitem not at all important	○	○	○	○	○	essential

Unable to save your draft

Does your paper address subitem 5-ii?

Copy and paste relevant sections from the manuscript (include quotes in quotation marks "like this" to indicate direct quotes from your manuscript), or elaborate on this item by providing additional information not in the ms, or briefly explain why the item is not applicable/relevant for your study

"We designed an technology-based family-centered goal-setting tool (GoalKeeper) using iterative feedback from parents and providers of CMC and informed by the challenges of technology-based tools elucidated in our prior work.[15] In the first phase of the design process we interviewed parents and providers to develop a taxonomy of goals and effective approaches to discussing goals in an outpatient clinic visit, based on prior frameworks for goal-concordant, chronic-illness care.[20-22] In the second phase, we conducted iterative user testing with parents and providers initially via paper-based prototypes and subsequently a technology-based version. We chose to host the intervention on the Internet and to function outside of the electronic health record (EHR) to allow for rapid iteration of the intervention design and ease of access to the intervention from any device (e.g. computer, tablet, smartphone). We designed GoalKeeper using responsive web technology, which optimizes its use for any device. This functionality enables parents and providers can quickly input data and access GoalKeeper in any setting with Internet independent of the type of device used."

5-iii) Revisions and updating

Revisions and updating. Clearly mention the date and/or version number of the application/intervention (and comparator, if applicable) evaluated, or describe whether the intervention underwent major changes during the evaluation process, or whether the development and/or content was “frozen” during the trial. Describe dynamic components such as news feeds or changing content which may have an impact on the replicability of the intervention (for unexpected events see item 3b).

	1	2	3	4	5	
subitem not at all important	○	○	○	○	○	essential

Does your paper address subitem 5-iii?

Copy and paste relevant sections from the manuscript (include quotes in quotation marks "like this" to indicate direct quotes from your manuscript), or elaborate on this item by providing additional information not in the ms, or briefly explain why the item is not applicable/relevant for your study

We have added the following, "During the trial, further development was frozen to ensure all participants were exposed to the same version of the intervention."

Unable to save your draft

5-iv) Quality assurance methods

Provide information on quality assurance methods to ensure accuracy and quality of information provided [1], if applicable.

	1	2	3	4	5	
subitem not at all important	○	○	○	○	○	essential

Does your paper address subitem 5-iv?

Copy and paste relevant sections from the manuscript (include quotes in quotation marks "like this" to indicate direct quotes from your manuscript), or elaborate on this item by providing additional information not in the ms, or briefly explain why the item is not applicable/relevant for your study

"The intervention implementation process was developed and evaluated using the Consolidated Framework for Implementation Research (CFIR).[24] Prior to the start of the trial, we conducted stakeholder focus groups using the CFIR companion interview guide to inform implementation of GoalKeeper that resulted in the selection of three implementation strategies: educational materials, individual technical assistance, and automated reminders.[25] Educational materials including paper and electronic copies of an instructional manual on GoalKeeper, with parent and provider versions. Parents received a 5-min in person overview of GoalKeeper while providers received a 30-min orientation, including a 5-min educational video.[26] The study team was available for in-person and remote technical assistance but did not attend clinic visits with participants. Parents received automated weekly e-mail reminders to track their progress on goals. Details on the implementation process and evaluation can be found in a companion manuscript.[27]"

5-v) Ensure replicability by publishing the source code, and/or providing screenshots/screen-capture video, and/or providing flowcharts of the algorithms used

Ensure replicability by publishing the source code, and/or providing screenshots/screen-capture video, and/or providing flowcharts of the algorithms used. Replicability (i.e., other researchers should in principle be able to replicate the study) is a hallmark of scientific reporting.

	1	2	3	4	5	
subitem not at all important	○	○	○	○	○	essential

Does your paper address subitem 5-v?

Copy and paste relevant sections from the manuscript (include quotes in quotation marks "like this" to indicate direct quotes from your manuscript), or elaborate on this item by providing additional information not in the ms, or briefly explain why the item is not applicable/relevant for your study

Figure 2 includes screenshots from the tool.

Unable to save your draft

5-vi) Digital preservation

Digital preservation: Provide the URL of the application, but as the intervention is likely to change or disappear over the course of the years; also make sure the intervention is archived (Internet Archive, webcitation.org, and/or publishing the source code or screenshots/videos alongside the article). As pages behind login screens cannot be archived, consider creating demo pages which are accessible without login.

	1	2	3	4	5	
subitem not at all important	○	○	○	○	○	essential

Does your paper address subitem 5-vi?

Copy and paste relevant sections from the manuscript (include quotes in quotation marks "like this" to indicate direct quotes from your manuscript), or elaborate on this item by providing additional information not in the ms, or briefly explain why the item is not applicable/relevant for your study

The intervention is no longer available due to subsequent concerns for critically needed updates to privacy and security from the hosting institution that resulted in their recommendation that the tool be taken down.

5-vii) Access

Access: Describe how participants accessed the application, in what setting/context, if they had to pay (or were paid) or not, whether they had to be a member of specific group. If known, describe how participants obtained "access to the platform and Internet" [1]. To ensure access for editors/reviewers/readers, consider to provide a "backdoor" login account or demo mode for reviewers/readers to explore the application (also important for archiving purposes, see vi).

	1	2	3	4	5	
subitem not at all important	○	○	○	○	○	essential

Does your paper address subitem 5-vii? *

Copy and paste relevant sections from the manuscript (include quotes in quotation marks "like this" to indicate direct quotes from your manuscript), or elaborate on this item by providing additional information not in the ms, or briefly explain why the item is not applicable/relevant for your study

We have added the following, "Participants in the intervention group were given GoalKeeper to use during their initial clinic visit immediately after enrolling in the study. They created a unique login and password and were emailed the the webpage link to access GoalKeeper on their own device. Study team members also accessed the GoalKeeper webpage on the clinic room computer immediately prior to the start of the participant's clinic visit."

Unable to save your draft

5-viii) Mode of delivery, features/functionalities/components of the intervention and comparator, and the theoretical framework

Describe mode of delivery, features/functionalities/components of the intervention and comparator, and the theoretical framework [6] used to design them (instructional strategy [1], behaviour change techniques, persuasive features, etc., see e.g., [7, 8] for terminology). This includes an in-depth description of the content (including where it is coming from and who developed it) [1]," whether [and how] it is tailored to individual circumstances and allows users to track their progress and receive feedback" [6]. This also includes a description of communication delivery channels and – if computer-mediated communication is a component – whether communication was synchronous or asynchronous [6]. It also includes information on presentation strategies [1], including page design principles, average amount of text on pages, presence of hyperlinks to other resources, etc. [1].

	1	2	3	4	5	
subitem not at all important	○	○	○	○	○	essential

Unable to save your draft

Does your paper address subitem 5-viii? *

Copy and paste relevant sections from the manuscript (include quotes in quotation marks "like this" to indicate direct quotes from your manuscript), or elaborate on this item by providing additional information not in the ms, or briefly explain why the item is not applicable/relevant for your study

For the intervention group, we described the functions of the tool as follows: "GoalKeeper consists of two separate modules: 1) Goal Elicitation and 2) Tracking. The key module and focus of this study is the Goal Elicitation module, which was designed to be used in real time in the exam room, facilitating a conversation between the parent and the medical provider during a routine, non-urgent care visit, consists of three components, as shown in Figure 2. The first component provides prompts to elicit the parent's "wishes/worries/ concerns" about their child's health. The second component helps the parent and provider derive from these "wishes/worries/concerns" at least one goal that is specific, measurable, and timebound to ensure that the goals were actionable and attainable. Specific actions were not entered into the module to reduce documentation burden based on feedback during design interviews and pre-testing. The third component provides sample goals as inspiration based on a taxonomy of common CMC goals developed in the design process. [23] These three components are synthesized on final screen, with a "copy" button to facilitate easy transfer (pasting) of the text into the EHR. The Tracking module was designed to be set-up by a provider immediately after the clinical encounter. The purpose of the Tracking module was to address parents' desires for symptoms tracking in the tool but was not the focus of the GoalKeeper design process given the prevalence of symptom tracking technology already in existence. The Tracking module provides customizable templates that providers can assign to parents to track their progress toward the elicited goal(s). To allow for asynchronous work outside of clinic visits, parents and providers had distinct interfaces. Providers could create goals, create and assign tracking templates, and view data while parents could view set goals and view and input data into tracking templates. We placed Goal Elicitation in the provider interface, based on user feedback, to help flatten the parent-provider hierarchy in the clinic visit. GoalKeeper requires the medical provider to elicit and type the parent's stated goals during the clinic visit. Prior prototypes that required parents to type their goals resulted in increased parental stress and decreased medical provider engagement. In contrast to usual practice, medical providers welcomed the during-encounter Goal Elicitation feature as an opportunity to engage more meaningfully with parents. We optimized the Tracking module for use on smartphones or tablets based on parent feedback. During the trial, further development was frozen to ensure all participants were exposed to the same version of the intervention."
For the control group, we described usual care as follows: "Participants in the control group received usual clinical care with their provider."

5-ix) Describe use parameters

Describe use parameters (e.g., intended "doses" and optimal timing for use). Clarify what instructions or recommendations were given to the user, e.g., regarding timing, frequency, heaviness of use, if any, or was the intervention used ad libitum.

	1	2	3	4	5	
subitem not at all important	○	○	○	○	○	essential

Unable to save your draft

Does your paper address subitem 5-ix?

Copy and paste relevant sections from the manuscript (include quotes in quotation marks "like this" to indicate direct quotes from your manuscript), or elaborate on this item by providing additional information not in the ms, or briefly explain why the item is not applicable/relevant for your study

"Parents received automated weekly e-mail reminders to track their progress on goals. Details on the implementation process and evaluation can be found in a companion manuscript.[27]"

5-x) Clarify the level of human involvement

Clarify the level of human involvement (care providers or health professionals, also technical assistance) in the e-intervention or as co-intervention (detail number and expertise of professionals involved, if any, as well as “type of assistance offered, the timing and frequency of the support, how it is initiated, and the medium by which the assistance is delivered”. It may be necessary to distinguish between the level of human involvement required for the trial, and the level of human involvement required for a routine application outside of a RCT setting (discuss under item 21 – generalizability).

	1	2	3	4	5	
subitem not at all important	○	○	○	○	○	essential

Does your paper address subitem 5-x?

Copy and paste relevant sections from the manuscript (include quotes in quotation marks "like this" to indicate direct quotes from your manuscript), or elaborate on this item by providing additional information not in the ms, or briefly explain why the item is not applicable/relevant for your study

"Prior to the start of the trial, we conducted stakeholder focus groups using the CFIR companion interview guide to inform implementation of GoalKeeper that resulted in the selection of three implementation strategies: educational materials, individual technical assistance, and automated reminders.[25] Educational materials including paper and electronic copies of an instructional manual on GoalKeeper, with parent and provider versions. Parents received a 5-min in person overview of GoalKeeper while providers received a 30-min orientation, including a 5-min educational video.[26] The study team was available for in-person and remote technical assistance but did not attend clinic visits with participants. Parents received automated weekly e-mail reminders to track their progress on goals. "
"Participants in the intervention group were given GoalKeeper to use during their initial clinic visit immediately after enrolling in the study. They created a unique login and password and were emailed the the webpage link to access GoalKeeper on their own device. Study team members also accessed the GoalKeeper webpage on the clinic room computer immediately prior to the start of the participant’s clinic visit. "

Unable to save your draft

5-xi) Report any prompts/reminders used

Report any prompts/reminders used: Clarify if there were prompts (letters, emails, phone calls, SMS) to use the application, what triggered them, frequency etc. It may be necessary to distinguish between the level of prompts/reminders required for the trial, and the level of prompts/reminders for a routine application outside of a RCT setting (discuss under item 21 – generalizability).

	1	2	3	4	5	
subitem not at all important	○	○	○	○	○	essential

Does your paper address subitem 5-xi? *

Copy and paste relevant sections from the manuscript (include quotes in quotation marks "like this" to indicate direct quotes from your manuscript), or elaborate on this item by providing additional information not in the ms, or briefly explain why the item is not applicable/relevant for your study

"Parents received automated weekly e-mail reminders to track their progress on goals. "

5-xii) Describe any co-interventions (incl. training/support)

Describe any co-interventions (incl. training/support): Clearly state any interventions that are provided in addition to the targeted eHealth intervention, as ehealth intervention may not be designed as stand-alone intervention. This includes training sessions and support [1]. It may be necessary to distinguish between the level of training required for the trial, and the level of training for a routine application outside of a RCT setting (discuss under item 21 – generalizability.

	1	2	3	4	5	
subitem not at all important	○	○	○	○	○	essential

Does your paper address subitem 5-xii? *

Copy and paste relevant sections from the manuscript (include quotes in quotation marks "like this" to indicate direct quotes from your manuscript), or elaborate on this item by providing additional information not in the ms, or briefly explain why the item is not applicable/relevant for your study

"Parents received a 5-min in person overview of GoalKeeper while providers received a 30-min orientation, including a 5-min educational video.[26]"

6a) Completely defined pre-specified primary and secondary outcome measures, including how and when they were assessed

Unable to save your draft

Does your paper address CONSORT subitem 6a? *

Copy and paste relevant sections from the manuscript (include quotes in quotation marks "like this" to indicate direct quotes from your manuscript), or elaborate on this item by providing additional information not in the ms, or briefly explain why the item is not applicable/relevant for your study

"The primary outcome was the difference in parent-reported quality of goal-setting between the control and intervention groups from T1 to T2, as measured by adapted questions from the goal-setting domain of the Patients Assessment of Chronic Illness Care (PACIC), a 20-item (5-point Likert scale: no/never to yes/always) validated survey measure of chronic illness care quality based on the Chronic Care Model.[29] A mean composite score is calculated for each domain and the entire survey based on relevant items. The goal-setting domain contains 5 items that cover healthy behaviors, treatment plans, and community integration. We adapted this measure for CMC because of its wide use in assessing chronic illness care quality and the lack of alternate, validated, pediatric-specific measures. Secondary outcomes measures included the PACIC subdomains for problem solving, care coordination, and composite outcome of care quality based on all measured PACIC subdomains.
Additional secondary outcome measures included quality of shared decision making, quality of parent-provider communication, and parenting stress. We assessed quality of shared decision making (T1, T2) using the National Survey for Children with Special Health Care Needs 2009-2010 shared decision making domain, which creates a binary composite variable for high versus low quality shared decision making from four survey questions at T1 and T2.[30] We assessed quality of parent-provider communication using the Communication Assessment Tool (T1), a validated 15-item (5-point Likert scale: 1-poor to 5-excellent) survey measure with mean composite outcome.[31] We assessed parenting stress using the Parenting Distress domain of the Parenting Stress Index (T1), which captures parent stress levels related to partner conflicts, social support, and life restrictions due to child rearing.[32] Scores are presented as a sum of all answers (range 12-60) with higher scores indicating higher stress. "

6a-i) Online questionnaires: describe if they were validated for online use and apply CHERRIES items to describe how the questionnaires were designed/deployed

If outcomes were obtained through online questionnaires, describe if they were validated for online use and apply CHERRIES items to describe how the questionnaires were designed/deployed [9].

	1	2	3	4	5	
subitem not at all important	○	○	○	○	○	essential

Unable to save your draft

Does your paper address subitem 6a-i?

Copy and paste relevant sections from manuscript text

This was a closed questionnaire for only those participants approached and consented by the study team. The outcomes were not validated for online use. The survey was administered through Stanford REDCap, with a unique identifier assigned to each participant. Survey administration methods are described as follows: "Measures of primary and secondary outcomes were collected electronically immediately after the initial clinical encounter for both parents and providers following study enrollment (T1) and at 1-month post-encounter (T2). Additional implementation outcome survey measures and exit interviews were collected at 3-months post-encounter (T3) for parents and, for providers, at the end of study period for their last enrolled parent. (See Supplemental Figure 3.) All measures were collected via secure electronic survey tools and stored electronically via the Stanford REDCap. "

6a-ii) Describe whether and how “use” (including intensity of use/dosage) was defined/measured/monitored

Describe whether and how “use” (including intensity of use/dosage) was defined/measured/monitored (logins, logfile analysis, etc.). Use/adoption metrics are important process outcomes that should be reported in any ehealth trial.

	1	2	3	4	5	
subitem not at all important	○	○	○	○	○	essential

Does your paper address subitem 6a-ii?

Copy and paste relevant sections from manuscript text

"We assessed intervention fidelity and quality in two ways: 1) capture of information directly from the GoalKeeper platform and 2) participant interviews at T3. Within the application, we primarily assessed number of goals set per intervention group participant but also assessed frequency of application use, and counts of data entered into each field. Through semi-structured interviews, we assessed barriers and facilitators to implementation utilizing questions from the CFIR companion interview guide, detailed in a separate manuscript.[27, 33]"

6a-iii) Describe whether, how, and when qualitative feedback from participants was obtained

Describe whether, how, and when qualitative feedback from participants was obtained (e.g., through emails, feedback forms, interviews, focus groups).

	1	2	3	4	5	
subitem not at all important	○	○	○	○	○	essential

Unable to save your draft

Does your paper address subitem 6a-iii?

Copy and paste relevant sections from manuscript text

"Through semi-structured interviews, we assessed barriers and facilitators to implementation utilizing questions from the CFIR companion interview guide, detailed in a separate manuscript.[27, 33]"

6b) Any changes to trial outcomes after the trial commenced, with reasons

Does your paper address CONSORT subitem 6b? *

Copy and paste relevant sections from the manuscript (include quotes in quotation marks "like this" to indicate direct quotes from your manuscript), or elaborate on this item by providing additional information not in the ms, or briefly explain why the item is not applicable/relevant for your study

There were no changes to trial outcomes after the trial commenced.

7a) How sample size was determined

NPT: When applicable, details of whether and how the clustering by care provides or centers was addressed

7a-i) Describe whether and how expected attrition was taken into account when calculating the sample size

Describe whether and how expected attrition was taken into account when calculating the sample size.

	1	2	3	4	5	
subitem not at all important	○	○	○	○	○	essential

Does your paper address subitem 7a-i?

Copy and paste relevant sections from manuscript title (include quotes in quotation marks "like this" to indicate direct quotes from your manuscript), or elaborate on this item by providing additional information not in the ms, or briefly explain why the item is not applicable/relevant for your study

"With a sample size of 40 parents (20 controls, 20 intervention), we were powered to detect a statistically significant difference of 0.5 SD or 0.6 absolute change on the PACIC goal-setting domain. With a projected attrition rate of 33%, our targeted recruitment was 60 parents (30 controls, 30 intervention)."

Unable to save your draft

Does your paper address CONSORT subitem 7b? *

Copy and paste relevant sections from the manuscript (include quotes in quotation marks "like this" to indicate direct quotes from your manuscript), or elaborate on this item by providing additional information not in the ms, or briefly explain why the item is not applicable/relevant for your study

We did not have any interim analyses or stopping guidelines.

8a) Method used to generate the random allocation sequence

NPT: When applicable, how care providers were allocated to each trial group

Does your paper address CONSORT subitem 8a? *

Copy and paste relevant sections from the manuscript (include quotes in quotation marks "like this" to indicate direct quotes from your manuscript), or elaborate on this item by providing additional information not in the ms, or briefly explain why the item is not applicable/relevant for your study

"The order that providers crossed-over was randomized in R (JLL) with allocation and enrollment by research assistants.[16]"

8b) Type of randomisation; details of any restriction (such as blocking and block size)

Does your paper address CONSORT subitem 8b? *

Copy and paste relevant sections from the manuscript (include quotes in quotation marks "like this" to indicate direct quotes from your manuscript), or elaborate on this item by providing additional information not in the ms, or briefly explain why the item is not applicable/relevant for your study

"In the stepped-wedge design, each provider first spent at least 3 weeks in the control condition, when research staff recruited parent-child dyads from their clinic to be in the control group, followed by cross-over into the intervention group for 3 weeks. Providers crossed-over at different intervals spaced 3 weeks apart. (See Supplemental Figure 1.) "

9) Mechanism used to implement the random allocation sequence (such as sequentially numbered containers), describing any steps taken to conceal the sequence until interventions were assigned

Unable to save your draft

Does your paper address CONSORT subitem 9? *

Copy and paste relevant sections from the manuscript (include quotes in quotation marks "like this" to indicate direct quotes from your manuscript), or elaborate on this item by providing additional information not in the ms, or briefly explain why the item is not applicable/relevant for your study

"The order that providers crossed-over was randomized in R (JLL) with allocation and enrollment by research assistants.[16]"

10) Who generated the random allocation sequence, who enrolled participants, and who assigned participants to interventions

Does your paper address CONSORT subitem 10? *

Copy and paste relevant sections from the manuscript (include quotes in quotation marks "like this" to indicate direct quotes from your manuscript), or elaborate on this item by providing additional information not in the ms, or briefly explain why the item is not applicable/relevant for your study

"The order that providers crossed-over was randomized in R (JLL) with allocation and enrollment by research assistants.[16]"

11a) If done, who was blinded after assignment to interventions (for example, participants, care providers, those assessing outcomes) and how

NPT: Whether or not administering co-interventions were blinded to group assignment

11a-i) Specify who was blinded, and who wasn't

Specify who was blinded, and who wasn't. Usually, in web-based trials it is not possible to blind the participants [1, 3] (this should be clearly acknowledged), but it may be possible to blind outcome assessors, those doing data analysis or those administering co-interventions (if any).

	1	2	3	4	5	
subitem not at all important	○	○	○	○	○	essential

Does your paper address subitem 11a-i? *

Copy and paste relevant sections from the manuscript (include quotes in quotation marks "like this" to indicate direct quotes from your manuscript), or elaborate on this item by providing additional information not in the ms, or briefly explain why the item is not applicable/relevant for your study

We did not have blinding in the study.

Unable to save your draft

11a-ii) Discuss e.g., whether participants knew which intervention was the "intervention of interest" and which one was the "comparator"

Informed consent procedures (4a-ii) can create biases and certain expectations - discuss e.g., whether participants knew which intervention was the "intervention of interest" and which one was the "comparator".

	1	2	3	4	5	
subitem not at all important	○	○	○	○	○	essential

Does your paper address subitem 11a-ii?

Copy and paste relevant sections from the manuscript (include quotes in quotation marks "like this" to indicate direct quotes from your manuscript), or elaborate on this item by providing additional information not in the ms, or briefly explain why the item is not applicable/relevant for your study

Participants in the intervention group knew the intervention they received as the intervention of interest. Participants in the control group knew they were receiving usual care.

11b) If relevant, description of the similarity of interventions

(this item is usually not relevant for ehealth trials as it refers to similarity of a placebo or sham intervention to a active medication/intervention)

Does your paper address CONSORT subitem 11b? *

Copy and paste relevant sections from the manuscript (include quotes in quotation marks "like this" to indicate direct quotes from your manuscript), or elaborate on this item by providing additional information not in the ms, or briefly explain why the item is not applicable/relevant for your study

There was only one intervention, so there is no description of the similarity between usual care and the intervention.

12a) Statistical methods used to compare groups for primary and secondary outcomes

NPT: When applicable, details of whether and how the clustering by care providers or centers was addressed

Unable to save your draft

Does your paper address CONSORT subitem 12a? *

Copy and paste relevant sections from the manuscript (include quotes in quotation marks "like this" to indicate direct quotes from your manuscript), or elaborate on this item by providing additional information not in the ms, or briefly explain why the item is not applicable/relevant for your study

"For primary and secondary outcome measures, we used intention-to-treat analysis with repeated measures ANOVA to evaluate for between-group and within-group differences in the PACIC domain of goal-setting for intervention versus control group while adjusting for: fixed-effects: timing of measurement (1-immediately after the visit, 2-1 month after the visit) and intervention-by-time interaction, as well as random effects: provider cluster. We used a similar approach for our secondary outcomes...We used a statistical significance level of p<0.05."

12a-i) Imputation techniques to deal with attrition / missing values

Imputation techniques to deal with attrition / missing values: Not all participants will use the intervention/comparator as intended and attrition is typically high in ehealth trials. Specify how participants who did not use the application or dropped out from the trial were treated in the statistical analysis (a complete case analysis is strongly discouraged, and simple imputation techniques such as LOCF may also be problematic [4]).

	1	2	3	4	5	
subitem not at all important	○	○	○	○	○	essential

Does your paper address subitem 12a-i? *

Copy and paste relevant sections from the manuscript (include quotes in quotation marks "like this" to indicate direct quotes from your manuscript), or elaborate on this item by providing additional information not in the ms, or briefly explain why the item is not applicable/relevant for your study

We did not use imputation for missing values/attrition.

12b) Methods for additional analyses, such as subgroup analyses and adjusted analyses

Unable to save your draft

Does your paper address CONSORT subitem 12b? *

Copy and paste relevant sections from the manuscript (include quotes in quotation marks "like this" to indicate direct quotes from your manuscript), or elaborate on this item by providing additional information not in the ms, or briefly explain why the item is not applicable/relevant for your study

"We conducted post hoc analyses of outcome variables with adjustment for population characteristics with statistically significant differences between subgroups. For baseline characteristics and feasibility outcome measures, we conducted descriptive analyses using Fisher's exact test for binary and categorical variables and Wilcoxon sign-rank test for continuous variables. We used a statistical significance level of p<0.05."

X26) REB/IRB Approval and Ethical Considerations [recommended as subheading under "Methods"] (not a CONSORT item)

X26-i) Comment on ethics committee approval

	1	2	3	4	5	
subitem not at all important	○	○	○	○	○	essential

Does your paper address subitem X26-i?

Copy and paste relevant sections from the manuscript (include quotes in quotation marks "like this" to indicate direct quotes from your manuscript), or elaborate on this item by providing additional information not in the ms, or briefly explain why the item is not applicable/relevant for your study

"This study was approved by the Stanford Single Institutional Review Board (Protocol 38365"

x26-ii) Outline informed consent procedures

Outline informed consent procedures e.g., if consent was obtained offline or online (how? Checkbox, etc.?), and what information was provided (see 4a-ii). See [6] for some items to be included in informed consent documents.

	1	2	3	4	5	
subitem not at all important	○	○	○	○	○	essential

Unable to save your draft

Does your paper address subitem X26-ii?

Copy and paste relevant sections from the manuscript (include quotes in quotation marks "like this" to indicate direct quotes from your manuscript), or elaborate on this item by providing additional information not in the ms, or briefly explain why the item is not applicable/relevant for your study

"All participants gave documented informed consent prior to enrolling in the study. All potential participants had the option to opt out of the study if they were not interested. Provider participants were approached at routine division meetings when the study team presented the study, reviewed the consent form, and gave ample time for questions. Providers could provide consent during or after the team meetings. Potential parent participants were approached in-person in the waiting room for consent and enrollment prior to their clinic visit with a participating provider. We did not use remote recruitment due to concerns of intervention fidelity as GoalKeeper was designed to be used jointly with parents and providers during a clinic visit. Study team members presented the study, reviewed the consent form, and gave ample time for questions. Consent was obtained prior to the start of the clinic visit. "

X26-iii) Safety and security procedures

Safety and security procedures, incl. privacy considerations, and any steps taken to reduce the likelihood or detection of harm (e.g., education and training, availability of a hotline)

	1	2	3	4	5	
subitem not at all important	○	○	○	○	○	essential

Does your paper address subitem X26-iii?

Copy and paste relevant sections from the manuscript (include quotes in quotation marks "like this" to indicate direct quotes from your manuscript), or elaborate on this item by providing additional information not in the ms, or briefly explain why the item is not applicable/relevant for your study

"Each participant was assigned a randomly generated four digit unique identifier. The key linking the unique identifier to participant name and contact information was stored in a separate database."

RESULTS

13a) For each group, the numbers of participants who were randomly assigned, received intended treatment, and were analysed for the primary outcome

NPT: The number of care providers or centers performing the intervention in each group and the number of patients treated by each care provider in each center

Unable to save your draft

Does your paper address CONSORT subitem 13a? *

Copy and paste relevant sections from the manuscript (include quotes in quotation marks "like this" to indicate direct quotes from your manuscript), or elaborate on this item by providing additional information not in the ms, or briefly explain why the item is not applicable/relevant for your study

"We enrolled 67 parent-child dyads out of 124 eligible parents approached across 2 outpatient clinics and 11 medical providers. Of parent-child dyads, 32 were enrolled in the control group and 35 were enrolled in the intervention group. "

13b) For each group, losses and exclusions after randomisation, together with reasons

Does your paper address CONSORT subitem 13b? (NOTE: Preferably, this is shown in a CONSORT flow diagram) *

Copy and paste relevant sections from the manuscript (include quotes in quotation marks "like this" to indicate direct quotes from your manuscript), or elaborate on this item by providing additional information not in the ms, or briefly explain why the item is not applicable/relevant for your study

"During the study period, 10 parent-child dyads (2 control, 8 intervention) were lost to follow-up at T2, and 1 control group parent who was fostering a child with medical complexity was withdrawn due to the child being transferred out of their custody. We also had 2 providers lost to follow-up due to leaving the institution. (See CONSORT diagram in Figure 4). "

13b-i) Attrition diagram

Strongly recommended: An attrition diagram (e.g., proportion of participants still logging in or using the intervention/comparator in each group plotted over time, similar to a survival curve) or other figures or tables demonstrating usage/dose/engagement.

	1	2	3	4	5	
subitem not at all important	○	○	○	○	○	essential

Does your paper address subitem 13b-i?

Copy and paste relevant sections from the manuscript or cite the figure number if applicable (include quotes in quotation marks "like this" to indicate direct quotes from your manuscript), or elaborate on this item by providing additional information not in the ms, or briefly explain why the item is not applicable/relevant for your study

CONSORT diagram provided in Figure 4.

14a) Dates defining the periods of recruitment and follow-up

Unable to save your draft

Does your paper address CONSORT subitem 14a? *

Copy and paste relevant sections from the manuscript (include quotes in quotation marks "like this" to indicate direct quotes from your manuscript), or elaborate on this item by providing additional information not in the ms, or briefly explain why the item is not applicable/relevant for your study

"Enrollment occurred between 4/1/19-12/21/20 with follow-up through 3/21/21."

14a-i) Indicate if critical “secular events” fell into the study period

Indicate if critical “secular events” fell into the study period, e.g., significant changes in Internet resources available or “changes in computer hardware or Internet delivery resources”

	1	2	3	4	5	
subitem not at all important	○	○	○	○	○	essential

Does your paper address subitem 14a-i?

Copy and paste relevant sections from the manuscript (include quotes in quotation marks "like this" to indicate direct quotes from your manuscript), or elaborate on this item by providing additional information not in the ms, or briefly explain why the item is not applicable/relevant for your study

"Due to institutional restrictions prompted by the COVID-19 pandemic prohibiting in-person research activities that were necessary for study activities at enrollment, recruitment was paused between 3/13/20-7/20/20."

14b) Why the trial ended or was stopped (early)

Does your paper address CONSORT subitem 14b? *

Copy and paste relevant sections from the manuscript (include quotes in quotation marks "like this" to indicate direct quotes from your manuscript), or elaborate on this item by providing additional information not in the ms, or briefly explain why the item is not applicable/relevant for your study

"Enrollment occurred between 4/1/19-12/21/20 with follow-up through 3/21/21."

15) A table showing baseline demographic and clinical characteristics for each group

NPT: When applicable, a description of care providers (case volume, qualification, expertise, etc.) and centers (volume) in each group

Unable to save your draft

Does your paper address CONSORT subitem 15? *

Copy and paste relevant sections from the manuscript (include quotes in quotation marks "like this" to indicate direct quotes from your manuscript), or elaborate on this item by providing additional information not in the ms, or briefly explain why the item is not applicable/relevant for your study

Table 1 described demographic and clinical characteristics

15-i) Report demographics associated with digital divide issues

In ehealth trials it is particularly important to report demographics associated with digital divide issues, such as age, education, gender, social-economic status, computer/Internet/ehealth literacy of the participants, if known.

	1	2	3	4	5	
subitem not at all important	○	○	○	○	○	essential

Does your paper address subitem 15-i? *

Copy and paste relevant sections from the manuscript (include quotes in quotation marks "like this" to indicate direct quotes from your manuscript), or elaborate on this item by providing additional information not in the ms, or briefly explain why the item is not applicable/relevant for your study

Table 1 described demographic and clinical characteristics including education, gender, and insurance status.

16) For each group, number of participants (denominator) included in each analysis and whether the analysis was by original assigned groups

16-i) Report multiple “denominators” and provide definitions

Report multiple “denominators” and provide definitions: Report N’s (and effect sizes) “across a range of study participation [and use] thresholds” [1], e.g., N exposed, N consented, N used more than x times, N used more than y weeks, N participants “used” the intervention/comparator at specific pre-defined time points of interest (in absolute and relative numbers per group). Always clearly define “use” of the intervention.

	1	2	3	4	5	
subitem not at all important	○	○	○	○	○	essential

Unable to save your draft

Does your paper address subitem 16-i? *

Copy and paste relevant sections from the manuscript (include quotes in quotation marks "like this" to indicate direct quotes from your manuscript), or elaborate on this item by providing additional information not in the ms, or briefly explain why the item is not applicable/relevant for your study

Table 2 reports the denominators for the outcome measures analyzed.

16-ii) Primary analysis should be intent-to-treat

Primary analysis should be intent-to-treat, secondary analyses could include comparing only "users", with the appropriate caveats that this is no longer a randomized sample (see 18-i).

	1	2	3	4	5	
subitem not at all important	○	○	○	○	○	essential

Does your paper address subitem 16-ii?

Copy and paste relevant sections from the manuscript (include quotes in quotation marks "like this" to indicate direct quotes from your manuscript), or elaborate on this item by providing additional information not in the ms, or briefly explain why the item is not applicable/relevant for your study

"For primary and secondary outcome measures, we used intention-to-treat analysis with repeated measures ANOVA to evaluate for between-group and within-group differences in the PACIC domain of goal-setting for intervention versus control group while adjusting for: fixed-effects: timing of measurement (1-immediately after the visit, 2-1 month after the visit) and intervention-by-time interaction, as well as random effects: provider cluster. We used a similar approach for our secondary outcomes. We conducted post hoc analyses of outcome variables with adjustment for population characteristics with statistically significant differences between subgroups."

17a) For each primary and secondary outcome, results for each group, and the estimated effect size and its precision (such as 95% confidence interval)

Unable to save your draft

Does your paper address CONSORT subitem 17a? *

Copy and paste relevant sections from the manuscript (include quotes in quotation marks "like this" to indicate direct quotes from your manuscript), or elaborate on this item by providing additional information not in the ms, or briefly explain why the item is not applicable/relevant for your study

"For our primary outcome measure, quality of goal-setting, scores were lower for the intervention group at t1 when compared to the control group, 2.41 (SD 1.07) versus 2.57 (SD 1.06). At 1-month follow-up (t2) the reverse was true with scores of 2.36 (SD 1.12) for the intervention group versus 2.15 (SD 1.23) for the control group. Quality of goal-setting between t1 and t2 remained stable for the intervention group but decreased for the control group (delta 0.03 versus -0.43, F-statistic 3.52, p-value 0.06). Similar patterns exist for other PACIC outcome measures of problem solving, care coordination, and overall care quality (composite). For other secondary outcome measures, parenting distress at t1 was higher in the intervention group when compared to the control group (mean 27.9 versus 22.2, p-value 0.02). There were no significant group differences in parent report of quality of shared decision making or quality of doctor-patient communication. (See Table 2 and Figure 5). "

17a-i) Presentation of process outcomes such as metrics of use and intensity of use

In addition to primary/secondary (clinical) outcomes, the presentation of process outcomes such as metrics of use and intensity of use (dose, exposure) and their operational definitions is critical. This does not only refer to metrics of attrition (13-b) (often a binary variable), but also to more continuous exposure metrics such as "average session length". These must be accompanied by a technical description how a metric like a "session" is defined (e.g., timeout after idle time) [1] (report under item 6a).

	1	2	3	4	5	
subitem not at all important	○	○	○	○	○	essential

Unable to save your draft

Does your paper address subitem 17a-i?

Copy and paste relevant sections from the manuscript (include quotes in quotation marks "like this" to indicate direct quotes from your manuscript), or elaborate on this item by providing additional information not in the ms, or briefly explain why the item is not applicable/relevant for your study

"Among the intervention group, 34 of 35 parents successfully used GoalKeeper with their provider to set goals during the clinic visit. For one parent, the provider decided during the visit that it was not an appropriate time to use GoalKeeper, but to preserve an intention-to-treat approach, that parent remained in the intervention group to complete the survey measures. Parents and providers set a median of 2.5 goals (range 0-4) per initial clinic visit, and providers assigned a median of 3 tracking templates (range 0-5) to each parent. Each provider saw a median of 2.5 parent participants (range 0-10). After the initial clinic visit, parents input information into GoalKeeper a median of 0 times (range 0-19) and providers viewed tracked data a median of 0.5 times throughout the study period (range 0-2). 19 parents (8 CPCC, 11 neurology) did not enter any data into the tracking templates while 13 parents (4 CPCC, 9 neurology) entered data 1-10 times, and 3 parents all from CPCC entered data over 10 times. In total, 87 goals were set with the most common domains being development (10 parents, 16 unique goals), child nutrition (13 parents, 13 unique goals), and seizure control (10 parents, 10 unique goals). There were no statistically significant differences in participant and patient characteristics between those who used the Tracking module more extensively than those who did not. Of the 19 parents who completed the exit survey, 13 parents (68%) would recommend GoalKeeper to other parents. 6 providers (67%) would recommend GoalKeeper to other providers. (See Supplemental Table 5.) "

17b) For binary outcomes, presentation of both absolute and relative effect sizes is recommended

Does your paper address CONSORT subitem 17b? *

Copy and paste relevant sections from the manuscript (include quotes in quotation marks "like this" to indicate direct quotes from your manuscript), or elaborate on this item by providing additional information not in the ms, or briefly explain why the item is not applicable/relevant for your study

We did not have binary outcomes.

18) Results of any other analyses performed, including subgroup analyses and adjusted analyses, distinguishing pre-specified from exploratory

Does your paper address CONSORT subitem 18? *

Copy and paste relevant sections from the manuscript (include quotes in quotation marks "like this" to indicate direct quotes from your manuscript), or elaborate on this item by providing additional information not in the ms, or briefly explain why the item is not applicable/relevant for your study

"For post-hoc analyses of ventriculoperitoneal shunt, the sample size of 4 was too small for valid statistical analysis."

Unable to save your draft

18-i) Subgroup analysis of comparing only users

A subgroup analysis of comparing only users is not uncommon in ehealth trials, but if done, it must be stressed that this is a self-selected sample and no longer an unbiased sample from a randomized trial (see 16-iii).

	1	2	3	4	5	
subitem not at all important	○	○	○	○	○	essential

Does your paper address subitem 18-i?

Copy and paste relevant sections from the manuscript (include quotes in quotation marks "like this" to indicate direct quotes from your manuscript), or elaborate on this item by providing additional information not in the ms, or briefly explain why the item is not applicable/relevant for your study

We did not perform such a subgroup analysis.

19) All important harms or unintended effects in each group

(for specific guidance see CONSORT for harms)

Does your paper address CONSORT subitem 19? *

Copy and paste relevant sections from the manuscript (include quotes in quotation marks "like this" to indicate direct quotes from your manuscript), or elaborate on this item by providing additional information not in the ms, or briefly explain why the item is not applicable/relevant for your study

There were no harms or unintended effects observed.

19-i) Include privacy breaches, technical problems

Include privacy breaches, technical problems. This does not only include physical “harm” to participants, but also incidents such as perceived or real privacy breaches [1], technical problems, and other unexpected/unintended incidents. “Unintended effects” also includes unintended positive effects [2].

	1	2	3	4	5	
subitem not at all important	○	○	○	○	○	essential

Unable to save your draft

Does your paper address subitem 19-i?

Copy and paste relevant sections from the manuscript (include quotes in quotation marks "like this" to indicate direct quotes from your manuscript), or elaborate on this item by providing additional information not in the ms, or briefly explain why the item is not applicable/relevant for your study

There were no privacy breaches or technical problems reported.

19-ii) Include qualitative feedback from participants or observations from staff/researchers

Include qualitative feedback from participants or observations from staff/researchers, if available, on strengths and shortcomings of the application, especially if they point to unintended/unexpected effects or uses. This includes (if available) reasons for why people did or did not use the application as intended by the developers.

	1	2	3	4	5	
subitem not at all important	○	○	○	○	○	essential

Does your paper address subitem 19-ii?

Copy and paste relevant sections from the manuscript (include quotes in quotation marks "like this" to indicate direct quotes from your manuscript), or elaborate on this item by providing additional information not in the ms, or briefly explain why the item is not applicable/relevant for your study

"In exit interviews, participants identified facilitators to GoalKeeper use to be the shared nature of the goal setting activity, the tool being accessible by the internet, and the ability to set long-term goals as facilitators to tool use. Per parent participants, the main barrier for sustained tool use after the initial goal-setting clinic visit was lack of integration in the electronic health record and patient portal. Additional details about the tool's feasibility and acceptability are reported in a companion manuscript.[27] "

DISCUSSION

22) Interpretation consistent with results, balancing benefits and harms, and considering other relevant evidence

NPT: In addition, take into account the choice of the comparator, lack of or partial blinding, and unequal expertise of care providers or centers in each group

Unable to save your draft

22-i) Restate study questions and summarize the answers suggested by the data, starting with primary outcomes and process outcomes (use)

Restate study questions and summarize the answers suggested by the data, starting with primary outcomes and process outcomes (use).

	1	2	3	4	5	
subitem not at all important	○	○	○	○	○	essential

Does your paper address subitem 22-i? *

Copy and paste relevant sections from the manuscript (include quotes in quotation marks "like this" to indicate direct quotes from your manuscript), or elaborate on this item by providing additional information not in the ms, or briefly explain why the item is not applicable/relevant for your study

"Our study demonstrated that the GoalKeeper intervention, a novel goal-setting platform for outpatient care of complex chronic illness, resulted in a better maintenance of overall chronic illness care quality, with no statistically significant impact on goal-setting, problem solving, or coordination. Intervention fidelity results indicate that the sustained effect of GoalKeeper on care quality was likely the result of participant engagement with the intervention's Goal Elicitation module, in contrast to its tracking module, which had very low participant engagement. Furthermore, we found that the intervention is both feasible and acceptable in the context of busy outpatient care, in both primary and subspecialty care settings. Together these results suggest that technology supports for goal-setting may be an effective way to maintain high quality care in between clinic visits for CMC but GoalKeeper itself needs further refinement to improve user experience. However, given the non-statistically significant findings in individual PACIC domains, the exact mechanism for this remains unclear. "

22-ii) Highlight unanswered new questions, suggest future research

Highlight unanswered new questions, suggest future research.

	1	2	3	4	5	
subitem not at all important	○	○	○	○	○	essential

Does your paper address subitem 22-ii?

Copy and paste relevant sections from the manuscript (include quotes in quotation marks "like this" to indicate direct quotes from your manuscript), or elaborate on this item by providing additional information not in the ms, or briefly explain why the item is not applicable/relevant for your study

"Nonetheless, given the non-statistically significant findings in individual PACIC domains, further research is necessary to extend and enhance the effectiveness of such platforms on care quality. " and "While our study demonstrates high initial adoption of GoalKeeper based on Goal Elicitation module use, future studies should investigate the mechanisms of effect and tools to sustain engagement, such as the effects of a more sophisticated Tracking module. "

Unable to save your draft

20) Trial limitations, addressing sources of potential bias, imprecision, and, if relevant, multiplicity of analyses

20-i) Typical limitations in ehealth trials

Typical limitations in ehealth trials: Participants in ehealth trials are rarely blinded. Ehealth trials often look at a multiplicity of outcomes, increasing risk for a Type I error. Discuss biases due to non-use of the intervention/usability issues, biases through informed consent procedures, unexpected events.

	1	2	3	4	5	
subitem not at all important	○	○	○	○	○	essential

Does your paper address subitem 20-i? *

Copy and paste relevant sections from the manuscript (include quotes in quotation marks "like this" to indicate direct quotes from your manuscript), or elaborate on this item by providing additional information not in the ms, or briefly explain why the item is not applicable/relevant for your study

"Study findings should be interpreted in the context of a few limitations. This study was conducted in a single children's health system, which may not be reflective of the practice at other institutions caring for CMC. However, the CPCC was part of a multi-center collaboration to develop care coordination programs for CMC, so some program similarities may exist across these collaborating sites. More participants in the intervention group were lost to follow-up, which may have introduced selection bias with those with poorer experiences with the intervention or with higher medical needs choosing not to continue to participate in the study. However, there were no statistically significant differences in parent and child characteristics between those lost to follow-up and those who completed the study. Notably participants had to have home Internet access, and while the divide between Internet access is narrowing, this could have excluded populations with lower household income.[44] The majority of our study participants also had at least some college education, which is reflective of the local population. Overall, our study period was quite short. Thus, the long-term intervention use, particularly on repeat clinic visit, was not observed. The effects of long-term intervention use should be assessed with future studies."

21) Generalisability (external validity, applicability) of the trial findings

NPT: External validity of the trial findings according to the intervention, comparators, patients, and care providers or centers involved in the trial

21-i) Generalizability to other populations

Generalizability to other populations: In particular, discuss generalizability to a general Internet population, outside of a RCT setting, and general patient population, including applicability of the study results for other organizations

	1	2	3	4	5	
subitem not at all [illegible]	○	○	○	○	○	essential

Unable to save your draft

Does your paper address subitem 21-i?

Copy and paste relevant sections from the manuscript (include quotes in quotation marks "like this" to indicate direct quotes from your manuscript), or elaborate on this item by providing additional information not in the ms, or briefly explain why the item is not applicable/relevant for your study

"Notably participants had to have home Internet access, and while the divide between Internet access is narrowing, this could have excluded populations with lower household income.[44] The majority of our study participants also had at least some college education, which is reflective of the local population."

21-ii) Discuss if there were elements in the RCT that would be different in a routine application setting

Discuss if there were elements in the RCT that would be different in a routine application setting (e.g., prompts/reminders, more human involvement, training sessions or other co-interventions) and what impact the omission of these elements could have on use, adoption, or outcomes if the intervention is applied outside of a RCT setting.

	1	2	3	4	5	
subitem not at all important	○	○	○	○	○	essential

Does your paper address subitem 21-ii?

Copy and paste relevant sections from the manuscript (include quotes in quotation marks "like this" to indicate direct quotes from your manuscript), or elaborate on this item by providing additional information not in the ms, or briefly explain why the item is not applicable/relevant for your study

"Overall, our study period was quite short. Thus, the long-term intervention use, particularly on repeat clinic visit, was not observed. The effects of long-term intervention use should be assessed with future studies."

OTHER INFORMATION

23) Registration number and name of trial registry

Does your paper address CONSORT subitem 23? *

Copy and paste relevant sections from the manuscript (include quotes in quotation marks "like this" to indicate direct quotes from your manuscript), or elaborate on this item by providing additional information not in the ms, or briefly explain why the item is not applicable/relevant for your study

"This study was approved by the Stanford Single Institutional Review Board (Protocol 38365, approved ***) and registered on clinicaltrials.gov (NCT03620071). "

Unable to save your draft

24) Where the full trial protocol can be accessed, if available

Does your paper address CONSORT subitem 24? *

Cite a Multimedia Appendix, other reference, or copy and paste relevant sections from the manuscript (include quotes in quotation marks "like this" to indicate direct quotes from your manuscript), or elaborate on this item by providing additional information not in the ms, or briefly explain why the item is not applicable/relevant for your study

"This study was approved by the Stanford Single Institutional Review Board (Protocol 38365, approved ***) and registered on clinicaltrials.gov (NCT03620071). "

25) Sources of funding and other support (such as supply of drugs), role of funders

Does your paper address CONSORT subitem 25? *

Copy and paste relevant sections from the manuscript (include quotes in quotation marks "like this" to indicate direct quotes from your manuscript), or elaborate on this item by providing additional information not in the ms, or briefly explain why the item is not applicable/relevant for your study

"Funding Statement: This work was supported by the National Institutes of Health, National Cancer Institute grant number R01CA204585. Dr. Lin received support from the National Institutes of Health, National Institute of Arthritis and Musculoskeletal and Skin Diseases 1K23AR079778. The funding sources had no involvement in the research activities related to this work."

X27) Conflicts of Interest (not a CONSORT item)

X27-i) State the relation of the study team towards the system being evaluated

In addition to the usual declaration of interests (financial or otherwise), also state the relation of the study team towards the system being evaluated, i.e., state if the authors/evaluators are distinct from or identical with the developers/sponsors of the intervention.

	1	2	3	4	5	
subitem not at all important	○	○	○	○	○	essential

Unable to save your draft

Does your paper address subitem X27-i?

Copy and paste relevant sections from the manuscript (include quotes in quotation marks "like this" to indicate direct quotes from your manuscript), or elaborate on this item by providing additional information not in the ms, or briefly explain why the item is not applicable/relevant for your study

"Conflicts of Interest: The authors have no competing interests to declare."

About the CONSORT EHEALTH checklist

As a result of using this checklist, did you make changes in your manuscript? *

- ○ yes, major changes
- ◉ yes, minor changes
- ○ no

What were the most important changes you made as a result of using this checklist?

Additional details in the methods around intervention deployment.

How much time did you spend on going through the checklist INCLUDING making changes in your manuscript *

I spent approximately two hours reviewing the checklist, making edits, and completing this form.

As a result of using this checklist, do you think your manuscript has improved? *

- ◉ yes
- ○ no
- ○ Other:

Unable to save your draft

Would you like to become involved in the CONSORT EHEALTH group?

This would involve for example becoming involved in participating in a workshop and writing an "Explanation and Elaboration" document

- ( ) yes
- (•) no
- ( ) Other:

Clear selection

Any other comments or questions on CONSORT EHEALTH

Your answer

STOP - Save this form as PDF before you click submit

To generate a record that you filled in this form, we recommend to generate a PDF of this page (on a Mac, simply select "print" and then select "print as PDF") before you submit it.

When you submit your (revised) paper to JMIR, please upload the PDF as supplementary file.

Don't worry if some text in the textboxes is cut off, as we still have the complete information in our database. Thank you!

Final step: Click submit !

Click submit so we have your answers in our database!

Submit   Clear form

Never submit passwords through Google Forms.

This content is neither created nor endorsed by Google. - Contact form owner - Terms of Service - Privacy Policy

Does this form look suspicious? Report

Google Forms

Unable to save your draft
